# *Peltigera* lichens as sources of uncharacterized cultured basidiomycete yeasts

**DOI:** 10.1186/s43008-024-00170-9

**Published:** 2024-12-04

**Authors:** Yosbany Pérez, Katerin Almendras, Ana M. Millanes, Nayla Serey, Andrey Yurkov, Natalia Lizana, Andrea Nesci, Aluminé Fessia, Julieta Orlando

**Affiliations:** 1 Instituto Milenio Biodiversidad de Ecosistemas Antárticos y Subantárticos (BASE), 7800003 Santiago, Chile; 2 Laboratorio de Ecología Microbiana, Departamento de Ciencias Ecológicas, Facultad de Ciencias, Universidad de Chile, 7800003 Santiago, Chile; 3 Departamento de Biología y Geología, Física y Química Inorgánica, Universidad Rey Juan Carlos (URJC), 28933 Móstoles, Spain; 4 Department of Bioresources for Bioeconomy and Health Research, Leibniz Institute DSMZ-German Collection of Microorganisms and Cell Cultures, Brunswick, Germany; 5 Laboratorio de Ecología Microbiana, Departamento de Microbiología E Inmunología, Facultad de Ciencias Exactas, Físico-Químicas y Naturales, Universidad Nacional de Río Cuarto, ICBIA (Instituto de Ciencias de La Tierra, Biodiversidad y Sustentabilidad Ambiental, CONICET-UNRC, Río Cuarto, Argentina; 6 Instituto de Investigación en Cambio Global (IICG-URJC), Universidad Rey Juan Carlos (URJC), 28933 Móstoles, Spain

**Keywords:** 4 new species, *Boekhoutia peltigerae*, *Cystobasidium chilense*, *Genolevuria patagonica*, *Pseudotremella navarinensis*, *Agaricostilbomycetes*, *Cystobasidiomycetes*, *Microbotryomycetes*, *Tremellomycetes*, Phylogeny

## Abstract

Lichens represent one of the most successful examples of symbiosis. They are constituted by the association between a dominant fungus (*i.e.*, the mycobiont), one or more photosynthetic partners (algae or cyanobacteria), and harbor an array of associated microorganisms, including bacteria and fungi. The associated fungal communities in lichens, known as the “lichen mycobiome”, are composed of both ascomycetes and basidiomycetes, including filamentous and yeast taxa. Recently, basidiomycete yeasts have received considerable attention as a much-overlooked source of diversity within the lichen mycobiome, with hypothesized roles in lichen symbiosis. This study surveyed the diversity of cultivable basidiomycete yeasts associated with *Peltigera* lichens across southern Chile. A phylogenetic study based on sequences of 179 yeast isolates allowed the identification of 29 taxa from 13 genera in the classes *Agaricostilbomycetes*, *Cystobasidiomycetes*, *Microbotryomycetes*, and *Tremellomycetes*, with the latter being the most represented. This research revealed several yeast species, including members of the genera *Boekhoutia and* *Goffeauzyma*, in lichens for the first time, thereby expanding our understanding of lichen-associated fungal diversity. In addition, four new cultivable species isolated from *Peltigera* are formally described. These are *Boekhoutia peltigerae* sp. nov., *Cystobasidium chilense* sp. nov., *Genolevuria patagonica* sp. nov. and *Pseudotremella navarinensis* sp. nov. These results highlight the role of lichens as reservoirs of uncharacterized basidiomycete yeasts.

**Supplementary Information:**

The online version contains supplementary material available at 10.1186/s43008-024-00170-9.

## Introduction

The definition of lichens has evolved considerably since Schwendener (1867), defined them as a dual symbiosis between a fungus and an alga. Recent advancements in lichen research have expanded our understanding of these organisms, now recognized as miniature self-sustaining ecosystems formed by the symbiotic association between a fungus (the primary mycobiont), extracellular microbial photosynthetic partners (an alga and/or a cyanobacterium), along with an indeterminate number of other associated microorganisms (Hawksworth and Grube 2020; Lücking et al. 2021). These symbiotic interactions lead to the formation of the lichen thallus, a unique symbiotic structure with extraordinary persistence and morphological variations that provide richly diverse and narrow-scale niches for microorganisms (Grube 2018). In this manner, lichens offer habitat and refuge for other organisms, supporting biological diversity (Zedda and Rambold 2015).

Besides the lichen mycobiont, other fungi frequently co-occur within lichen thalli, collectively known as the “lichen mycobiome”. This includes both ascomycetes and basidiomycetes, either filamentous (mycelium consisting of hyphae), yeast (unicellular), or dimorphic taxa, the later alternating a yeast and a filamentous stage in their life-cycle (Arnold et al. 2009; U’Ren et al. 2012; Fernández-Mendoza et al. 2017; Banchi et al. 2018; Diederich et al. 2018). Within the lichen mycobiome, basidiomycete yeasts have been reported to be regularly present in lichens (Spribille et al. 2016; Tuovinen et al. 2019, 2021; Tagirdzhanova et al. 2021). Yeasts belonging to the classes *Cystobasidiomycetes* and *Tremellomycetes* have been reported, including species in genera until now exclusively found in lichens (Prillinger et al. 1997; Park et al. 2015; Spribille et al. 2016; Pankratov et al. 2017; Černajová and Škaloud 2019; Tuovinen et al. 2019, 2021; Diederich et al. 2022). The presence of basidiomycete yeasts has been mainly studied in lichen species of the family *Parmeliaceae* and the genera *Lecanora* and *Cladonia* (Spribille et al. 2016; Černajová and Škaloud 2019; Tuovinen et al. 2019, 2021; Kachalkin et al. 2024), although other lichens have also been investigated (Prillinger et al. 1997; Cometto et al. 2022). It has been suggested that these basidiomycete yeasts may contribute to the formation of the shared extracellular matrix, participate in the production of secondary metabolites, and aid in phosphate solubilization (Spribille et al. 2016; Tagirdzhanova et al. 2021; da Silva et al. 2022a). However, the potential functions of the basidiomycete yeasts in the lichen symbiosis are still unclear, as well as how ubiquitous and specific these yeast asexual stages are (Fernández-Mendoza et al. 2017; Oberwinkler 2017; Lendemer et al. 2019; Mark et al. 2020). Our knowledge of the diversity of basidiomycete yeasts and their functions within lichen symbioses remains limited, with a high potential of revealing novel diversity within different lichens.

*Peltigera* is a genus of cosmopolitan lichens that includes muscicolous and terricolous foliose lichen-forming fungi mainly associated with cyanobacteria of the genus *Nostoc* (bipartite association), although a few species associate with green algae of the genus *Coccomyxa* as the main photobiont and *Nostoc* as the secondary photobiont (tripartite association) (Miadlikowska and Lutzoni 2000). In southern Chile, over ten bipartite *Peltigera* species have been reported, including cosmopolitan and endemic representatives (Magain et al. 2018; Miadlikowska et al. 2020, 2023; Orlando et al. 2023; Veas-Mattheos et al. 2023). These lichens grow in a diversity of habitats, such as *Nothofagus* forests, and in open spaces without forest cover, such as Patagonian and High Andean steppes, from both protected and unprotected areas (Zúñiga et al. 2015; Magain et al. 2018; Miadlikowska et al. 2020; Orlando et al. 2023; Veas-Mattheos et al. 2023). Recent findings have demonstrated that *Peltigera* lichens harbor a rich bacterial diversity that significantly enhances gamma diversity across landscapes, functioning as island-like habitats that support a potential reservoir of specialized bacteria (Schwob et al. 2024). In addition, the mycobiome of *Peltigera* lichens was reported as more diverse and specialized than that of *Parmelia* lichens, hosting a broader range of fungi and indicating a higher ecological resilience (Yang et al. 2024). However, the knowledge of yeasts in these lichens is limited. To date, *Rhodotorula mucilaginosa* is the only basidiomycete yeast isolated from *Peltigera* lichens collected in the Northern Hemisphere (Kachalkin et al. 2017), but representatives of basidiomycete yeasts in *Peltigera* lichens from the Southern Hemisphere remain unexplored.

The ecological diversity of *Peltigera* lichens in southern Chile, together with the diverse vegetation along the extensive latitudinal gradient and bioclimatic features that offer this region, provides an opportunity to uncover novel basidiomycete yeasts associated with lichen symbiosis. In this study, a culture-dependent approach was employed to analyze the phylogenetic diversity of basidiomycete yeasts associated with *Peltigera* lichens, including samples from protected and unprotected areas along a latitudinal gradient in southern Chile, expanding the taxonomic knowledge of the fungal communities associated with these lichens.

## Materials and methods

### Lichen samples and study sites

**Fig. 1:**
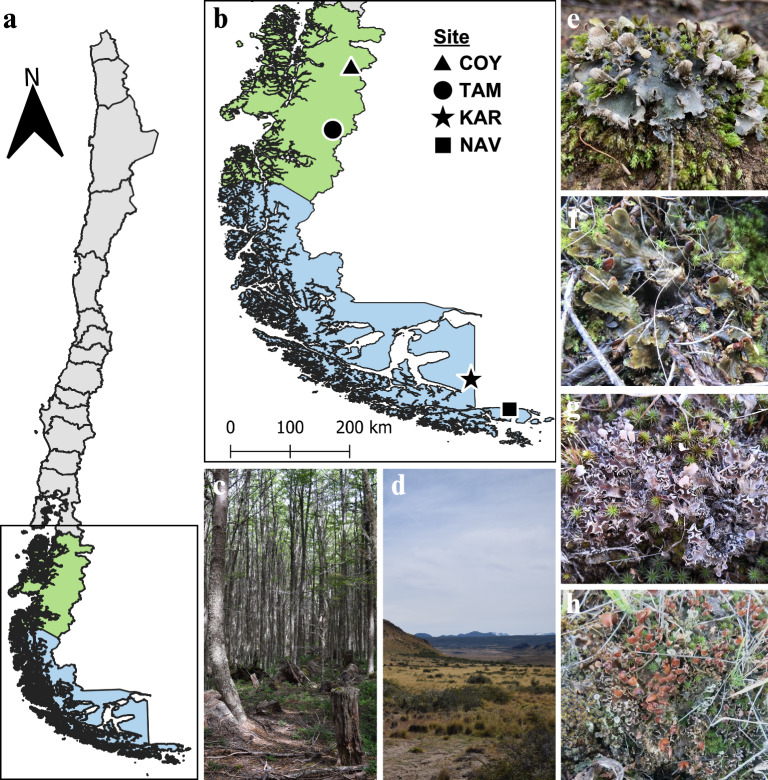
Map of Chile indicating the sampling regions (**a**). Distribution of the collection sites: Coyhaique National Reserve (COY), Patagonia National Park, Tamango area (TAM), Karukinka Natural Park (KAR), and Navarino Island (NAV) (**b**). Example of forest habitat type (**c**) and grassland habitat type (**d**). In addition, the lichen specimens collected from forests: *P. frigida* (**e**) and *P. “fuscopraetextata”* (**f**), and from grasslands: *P. rufescens* (**g**) and “*P.* *ponojensis/monticola* 11” (**h**) are shown. Map created using the free and open source QGIS

Fragments of *Peltigera* thalli were obtained from the *Peltigera* lichen collection of the Microbial Ecology Laboratory of Universidad de Chile (Orlando et al. 2023). Thalli, with no visible signs or structures indicating the presence of microparasites, were recovered from paper bags where they had been stored for varying periods. The oldest samples date back to 2013 and 2018, while the most recent ones were collected in 2019 and 2020, all kept at room temperature (Table 1). Four different *Peltigera* lichens were selected due to their predominance in various sites and different environments in southern Chile: *P. frigida*, *P.* “*fuscopraetextata*”, *P. rufescens*, and *Peltigera sp*. The last one is a still undescribed species referred to as “*P. ponojensis/monticola* 11” by Miadlikowska et al. (2023) and, for convenience, will be labeled that way in this work. These specimens were collected from forests and grasslands in four sites of southern Chile (Fig. 1): (i) Coyhaique National Reserve (hereinafter named Coyhaique), (ii) Patagonia National Park, Tamango area (hereinafter named Tamango), (iii) Karukinka Natural Park (hereinafter named Karukinka) and (iv) Navarino Island (hereinafter named Navarino). In Coyhaique, samples were taken from a young *Nothofagus pumilio* forest (651–710 m.a.s.l.) and a Patagonian steppe (668–706 m.a.s.l.). This area has an annual mean temperature of approximately 5.9 °C and receives around 1000 mm of precipitation yearly. In Tamango, samples were also gathered from a young *Nothofagus pumilio* forest (774–790 m.a.s.l.) and a Patagonian steppe (441–447 m.a.s.l.), where the mean annual temperature is about 6.1 °C, with yearly precipitation around 700 mm. In Karukinka, lichen samples came from a mature *Nothofagus pumilio* forest (158–164 m.a.s.l.) and an adjacent Patagonian steppe (154–168 m.a.s.l.), with an annual mean temperature of 5.0 °C and approximately 500 mm of rainfall. Finally, in Navarino, samples were taken from a mature *Nothofagus pumilio* forest (270–284 m.a.s.l.) and a nearby grassland (2–24 m.a.s.l.), where the mean annual temperature is 5.3 °C, and annual precipitation is around 530 mm. One thallus was selected from each lichen at each sampling site, resulting in 16 thallus samples from four different lichens. The identity of the lichens and information of the sites are shown in Table 1.

**Table 1:** *Peltigera* lichens and their geographic origin from which basidiomycete yeasts were isolated

Lichen ID	Lichen species	Habitat	Site	Region	Collection date	Latitude	Longitude	Altitude (m.a.s.l)
COY18-012	*Peltigera frigida*	*Nothofagus pumilio* young forest	Coyhaique	Aysen	2018–02–20	− 45.529475	− 72.028378	651.0
COY13-045	*Peltigera* “*fuscopraetextata*”	*Nothofagus pumilio* young forest	Coyhaique	Aysen	2013–02–22	− 45.529515	− 72.027832	710.2
COY19-007	“*Peltigera ponojensis/monticola* 11”	Patagonian steppe at the edge of a *Nothofagus pumilio* forest	Coyhaique	Aysen	2019–05–19	− 45.536316	− 72.019902	668.9
COY19-023	*Peltigera rufescens*	Patagonian steppe at the edge of a *Nothofagus pumilio* forest	Coyhaique	Aysen	2019–10–19	− 45.536205	− 72.020591	706.4
TAM19-020	*Peltigera frigida*	*Nothofagus pumilio* young forest	Tamango	Aysen	2019–02–20	− 47.214694	− 72.528395	774.2
TAM19-007	*Peltigera* “*fuscopraetextata*”	*Nothofagus pumilio* young forest	Tamango	Aysen	2019–02–20	− 47.214461	− 72.528428	790.9
TAM19-024	“*Peltigera ponojensis/monticola* 11”	Patagonian steppe	Tamango	Aysen	2019–02–20	− 47.221880	− 72.531300	441.4
TAM19-030	*Peltigera rufescens*	Patagonian steppe	Tamango	Aysen	2019–02–20	− 47.221660	− 72.531390	447.3
KAR20-045	*Peltigera frigida*	*Nothofagus pumilio* mature forest	Karukinka	Magallanes	2020–03–02	− 54.127038	− 68.709093	158.8
KAR20-065	*Peltigera* “*fuscopraetextata*”	*Nothofagus pumilio* mature forest	Karukinka	Magallanes	2020–03–02	− 54.126993	− 68.709751	164.0
KAR20-389	“*Peltigera ponojensis/monticola* 11”	Patagonian steppe	Karukinka	Magallanes	2020–03–06	− 54.126483	− 68.708319	168.5
KAR20-279	*Peltigera rufescens*	Patagonian steppe	Karukinka	Magallanes	2020–03–04	− 54.126438	− 68.708552	154.7
NAV20-110	*Peltigera frigida*	*Nothofagus pumilio* mature forest	Navarino	Magallanes	2020–02–23	− 54.954859	− 67.632293	284.9
NAV20-129	*Peltigera* “*fuscopraetextata*”	*Nothofagus pumilio* mature forest	Navarino	Magallanes	2020–02–23	− 54.954887	− 67.632822	270.2
NAV20-233	“*Peltigera ponojensis/monticola* 11”	Grassland	Navarino	Magallanes	2020–02–24	− 54.936232	− 67.655131	2.0
NAV20-214	*Peltigera rufescens*	Grassland	Navarino	Magallanes	2020–02–24	− 54.936346	− 67.655391	24.1

Approximately 0.05 g of each thallus was carefully taken using sterile pincers and placed in 15 ml Falcon tubes. Thalli underwent two wash steps with sterile distilled water and were agitated at 200 rpm for 10 min to remove microorganisms from the surface that were not closely associated with the lichen thallus. The washed fragments were then triturated in a sterile mortar with 3 ml of sterile distilled water under a burner. An aliquot of 100 µl of both undiluted and 1/10 (v/v) diluted crushed thalli was taken and spread on plates using a Drigalski spatula. The dilution step was employed to reduce the growth of filamentous fungi and enhance the yield of yeasts. Three distinct culture media were employed: (i) Yeast Malt Agar (YMA, 0.3% yeast extract, 0.3% malt extract, 0.5% peptone, 1.0% glucose, 1.5% agar) (Troncoso et al. 2017), (ii) Potato Dextrose Agar (PDA, Merck 110,130), and (iii) Yeast nitrogen base without amino acids (YNB without amino acids, Merck Y0626) supplemented with 2% glucose and 2% agar. Antibiotics (chloramphenicol and ampicillin at 100 µg/ml) were added to all media to inhibit bacterial growth. Then, six plates per sample (n = 96) were incubated at 10 °C in darkness for a maximum of 60 days with daily monitoring, as recommended by Santiago et al. (2015) for isolating fungi capable of growing and reproducing in cold temperatures. Colonies were then transferred to a fresh medium to optimize the recovery of yeast species. Filamentous fungi on the plates were carefully excised and discarded using a sterile scalpel to prevent interference with the growth of slow-growing yeasts. Isolates were preserved in cryotubes with sterile glycerol at a concentration of 15% and stored at − 20 °C. Additionally, the dehydrated gelatin drop method (DGD) at room temperature was employed for preservation (Marangon et al. 2003; Baeza et al. 2009). All isolates obtained in this study are part of the collection of basidiomycete yeasts isolated from *Peltigera* lichens at the Microbial Ecology Laboratory of the Universidad de Chile (Pérez et al. 2023).

### DNA extraction, PCR amplification, and sequencing

DNA extraction was conducted by obtaining small portions of the cultured yeast colonies with a sterile toothpick, which were then transferred into 0.2 ml tubes containing 10 µl of TE buffer (10 mM Tris, 1 mM EDTA, pH 8). The lysis process involved eight consecutive heat shock cycles, each consisting of 1 min at 98 °C followed by cooling to 4 °C, utilizing a Bio-Rad T100 Thermal Cycle (Bio-Rad, USA).

Subsequently, the ITS region was amplified using the ITS1F (Gardes and Bruns 1993) and ITS4 primers (White et al. 1990), and the LSU region using the LR0R and LR7 primers (Vilgalys and Hester 1990; De Long et al. 2013). PCR amplifications were conducted in a 25 µl reaction volume, containing 12.5 µl of GoTaq® Green Master Mix (Promega, USA), 0.5 µl of each primer at 10 µM, and 1 µl of yeast DNA, using a Bio-Rad T100 Thermal Cycle (Bio-Rad, USA). The PCR conditions for ITS amplification consisted of an initial denaturation of 5 min at 95 °C, followed by 30 cycles of 45 s at 95 °C, 30 s at 55 °C, 90 s at 72 °C, with a final extension of 10 min at 72 °C. For the amplification of the LSU region, the program included an initial denaturation at 95 °C for 3 min, followed by 40 cycles of 95 °C for 35 s, 47 °C for 1 min, and 72 °C for 2 min, with a final extension at 72 °C for 7 min.

The quality and size of the amplified products were assessed by agarose gel electrophoresis (2%, w/v) stained with GelRed (Biotium, USA). All amplicons were unidirectionally sequenced using the forward primer through a sequencing service provided by Macrogen in Seoul, South Korea, and Santiago, Chile. DNA sequences were visually checked and manually edited using SnapGene software (Insightful Science; http://www.snapgene.com) and Mega 11 software (Tamura et al. 2021). The ITS sequences obtained were deposited in the GenBank database with accession numbers OQ448325–OQ448503, while the LSU sequences were deposited under accession numbers PQ362932–PQ362992.

### Taxon sampling and phylogenetic analyses

Initially, the ITS and LSU sequences were compared with sequences in GenBank using BLASLn (Altschul et al. 1990). We performed the BLAST analysis exclusively against referenced sequences, using the standard database (nr) and fungal type sequences and reference material databases for each marker analyzed. The analysis was optimized for highly similar sequences (megablast), keeping all other parameters constant. The results retrieved by BLASTn showed that our sequences had high similarity with different representatives of three classes in the *Pucciniomycotina* (viz., *Agaricostilbomycetes*, *Cystobasidiomycetes,* and *Microbotryomycetes*) and with different representatives of the class *Tremellomycetes* (*Agaricomycotina*). Therefore, we constructed two initial LSU datasets for our isolates, one focusing on *Pucciniomycotina* and one on *Tremellomycetes*. When possible, sequences retrieved from GenBank included in the datasets were derived from type material. We used only LSU in these first two datasets, because the ITS1 and ITS2 regions were not alignable over this taxon sampling, and the 5.8S was uninformative.

Focused on the groups in which our isolates from *Peltigera* were placed in the phylogenetic analyses of the first two datasets, additional datasets for each group combining ITS and LSU sequences were performed. In some cases, we assembled ITS datasets to include sequences from isolates for which we could not obtain LSU sequences. Therefore, we assembled ITS datasets for genera *Filobasidium* (*Filobasidiaceae*, *Tremellomycetes*, *Agaricomycotina*), *Tausonia* (*Mrakiaceae*, *Tremellomycetes*, *Agaricomycotina*), *Solicoccozyma* (*Piskurozymaceae*, *Tremellomycetes*, *Agaricomycotina*) and *Vishniacozyma (Bulleribasidiaceae*, *Tremellomycetes*, *Agaricomycotina*), while combined ITS and LSU datasets for the order *Holtermanniales (Tremellomycetes, Agaricomycotina*), family *Chionosphaeraceae* (*Agaricostilbomycetes*, *Pucciniomycotina*), and genera *Cystobasidium* (*Cystobasidiaceae*, *Cystobasidiomycetes*, *Pucciniomycotina*), *Naganishia*, (*Filobasidiaceae*, *Tremellomycetes*, *Agaricomycotina*), *Genolevuria* (*Bulleraceae*, *Tremellomycetes*, *Agaricomycotina*), *Goffeauzyma* (*Filobasidiaceae*, *Tremellomycetes*, *Agaricomycotina*), *Pseudotremella* (*Bulleraceae, Tremellomycetes*, *Agaricomycotina*), *Rhodotorula* (*Sporidiobolaceae*, *Microbotryomycetes*, *Pucciniomycotina*) and *Teunia (Cryptococcaceae, Tremellomycetes, Agaricomycotina*). In these datasets, in addition to sequences derived from type material, we included a fair representation of the sequences more closely related to our isolates to achieve a more accurate species identification and to detect potential new taxa among our isolates from *Peltigera* (see information for each dataset in the corresponding figure legend).

Sequences were aligned using MAFFT version 7 (Katoh et al. 2018) with the Q-INS-i algorithm. The resulting alignments were then trimmed to eliminate ambiguously aligned regions. Both pre- and post-trimming checks were performed manually using the alignment viewer and editor AliView v1.27 (Larsson 2014). Gaps at the beginning and end of the alignments were treated as missing data. When applicable, each partition was analyzed individually using the maximum likelihood ultrafast bootstrap in IQTree to assess for potential topological conflicts. Strongly supported clades (IQTree UF-BS higher than 95%) in disagreement were considered an indication of a significant conflict (Mason-Gamer and Kellogg 1996; Hoang et al. 2018). Since no conflict was detected in our datasets, we combined and analyzed them using maximum likelihood.

Phylogenetic relationships were reconstructed employing Maximum likelihood (ML). The ML trees were generated using IQ-TREE software (Nguyen et al. 2015), and the substitution model was selected using the Akaike information criterion (AIC) or the corrected Akaike information criterion (AICc) through ModelFinder (Kalyaanamoorthy et al. 2017) implemented in IQ-TREE. Node support was evaluated by standard bootstrap with 1000 pseudo-replicates. A bootstrap percentage ≥ 70% was considered as a phylogenetic support. ML analyses were conducted using the CIPRES Science Gateway v.3.3 web portal (Miller et al. 2011) and IQ-TREE version 1.6.12 for Windows. Phylogenetic trees were visualized and edited in iTOL v6 (https://itol.embl.de).

### Phenotypic characterization

The cultures identified as potential new species through phylogenetic analyses were phenotypically characterized using standard methods described in Kurtzman et al. (2011) and Biolog’s YT MicroPlates™ (www.biolog.com) phenotypic technology for yeasts, for carbon-source assimilation tests.

To detect carbon dioxide production, glucose fermentation was assessed using 2% sugar in Durham tubes. We also evaluated the assimilation of 22 carbon sources (citrate, cellobiose, d-arabinose, l-arabinose, d-gluconate, d-mannitol, d-xylose, i-erythritol, ethanol, d-galactose, d-glucose, glycerol, meso-inositol, myo-inositol, lactose, l-rhamnose, maltose, melibiose, methanol, *N*-acetyl-d-glucosamine, d-raffinose, and sucrose) and five nitrogen sources (ammonium sulfate, creatine, creatinine, potassium nitrate, and sodium nitrite) in liquid media. Test tubes containing Yeast Nitrogen Base (Merck: 51,483) and Yeast Carbon Base (Merck: Y3627) were prepared with 1% of each carbon source and 0.108 g of nitrogen from each nitrogen source, respectively, following the protocol of Kurtzman et al. (2011). All tubes were inoculated with approximately 10⁷ cells/ml, as determined by Neubauer chamber counting. Growth was monitored by measuring optical density to 600 nm using a spectrophotometer (Bioteck, EpochTM) for up to 30 days at 15 °C. Carbon assimilation tests were also conducted using YT MicroPlate™, following the manufacturer’s recommendations with minor modifications. Each well was inoculated with 100 µl of yeast inoculum in sterile water at approximately 10⁷ cells/ml and incubated for 72 h at 15 °C. The presence of turbidity indicated the ability to assimilate carbon sources, and optical densities were measured at 600 nm.

The temperature range for yeast growth was evaluated using agar plates and liquid medium, both with YM medium. Growth was assessed visually on solid plates at various temperatures and 30 °C in a liquid medium. Optical density at 600 nm was measured for liquid cultures over five to 30 days.

The ability to produce extracellular amyloid compounds was investigated by plating on YM agar and inoculating tubes with 3 mL of liquid YM medium, both incubated in darkness at 15 °C for 2 weeks. Following incubation, Lugol’s iodine solution was added, and a color change to dark blue or green indicated the presence of amyloid compounds.

Yeast tolerance to high osmotic pressure was evaluated using a nitrogen base medium supplemented with 10% NaCl and 5% glucose, along with slanted agar containing 50% glucose and yeast extract, according to the protocol outlined by Kurtzman et al. (2011). Both were incubated at 15 °C in darkness for 30 days, measuring optical density at 600 nm for liquid cultures while observing agar growth visually.

To assess the ability to grow in a vitamin-free medium, cultures were incubated in a liquid medium according to Kurtzman et al. (2011) at 15 °C, and growth was measured by optical density after 30 days.

Micromorphological characterization of cells was performed on 5-day-old cultures grown in YMA supplemented with chloramphenicol at 15 °C. Cells were observed under a light microscope M10 model (Swift, USA) at 400 × magnification to determine microscopic characteristics, including cell shape and size, type of asexual reproduction, and the presence of germ tubes, pseudohyphae, and septate hyphae. Approximately 20 cells from each strain were measured, and the width-to-length ratio was calculated. For colony descriptions, color, surface texture, margin, elevation, shape, and size were assessed through direct observation using a stereoscopic microscope NE6745 model (Microimaging, Chile). Colony size was determined by averaging the width and length dimensions from five colonies of each isolated strain.

## Results

### Basidiomycete yeasts identification

A total of 179 basidiomycete yeasts were isolated from 15 *Peltigera* thalli (Table S1). The isolates were obtained from all samples except the *P.* “*fuscopraetextata*” thallus collected in Coyhaique in 2013 (sample COY13-045). The basidiomycete yeast cultures obtained in this study belonged to two subphyla, *Agaricomycotina* and *Pucciniomycotina*, encompassing four classes, seven orders, nine families, and 13 genera. First, two phylogenetic analyses were conducted at the subphylum *Pucciniomycotina* (Fig. S1) level and within the class *Tremellomycetes* (Fig. S2), based on LSU sequences, to determine the taxonomic placement of the isolates obtained in this study. Additionally, 13 separate phylogenetic analyses were performed to determine the identity of the isolates corresponding to the family *Chionosphaeraceae* (*Agaricostilbomycetes*) and the genera *Cystobasidium* (*Cystobasidiomycetes*), *Rhodotorula* (*Microbotryomycetes*), *Filobasidium*, *Teunia*, *Genolevuria*, *Goffeauzyma*, *Holtermanniella*, *Naganishia*, *Pseudotremella*, *Solicoccozyma*, *Tausonia*, and *Vishniacozyma* (*Tremellomycetes*).

### Class *Agaricostilbomycetes*

**Fig. 2:**
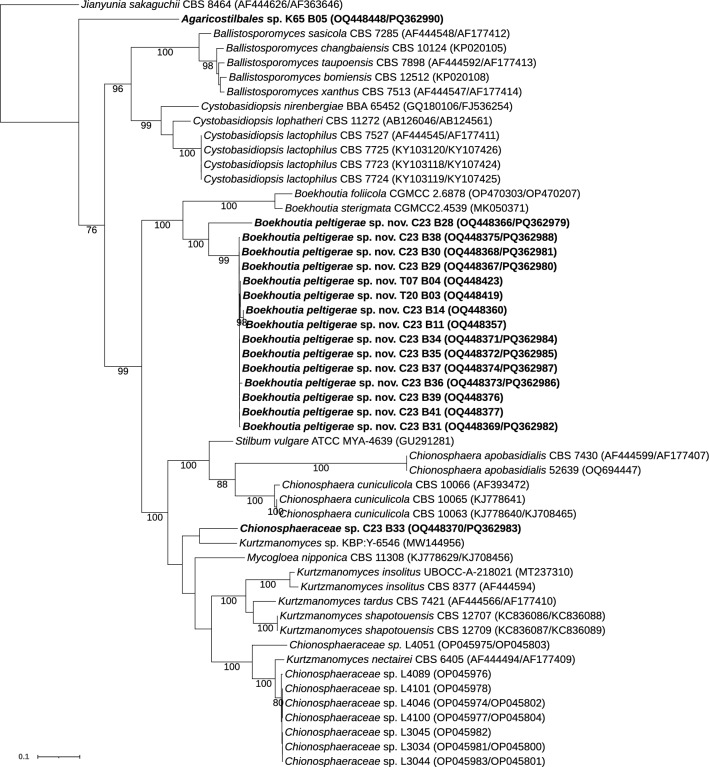
Phylogenetic relationships of yeast isolates obtained from *Peltigera* and related taxa in the family *Chionosphaeraceae* (*Agaricostilbomycetes*). The dataset included sequences representing the seven accepted genera in the family. *Jianyunia sakaguchii* was used as an outgroup based on Wang et al. (2015a). The alignment included 53 terminals with 1466 characters—565 of which were parsimony-informative and 732, constant. The substitution model GTR + F + I + G4 was selected for the ITS1, the TPM3 + FQ + G4 for the 5.8S, the HKY + F + I + G4 for the ITS2, and the TIM3 + F + I + G4 for the LSU. We considered four independent partitions, ITS1 (1–317), 5.8S (318–435), ITS2 (436–828) and LSU (829–1466). Maximum likelihood bootstrap values ≥ 70% are indicated below branches. The isolates obtained in this work are highlighted in bold

Within the class *Agaricostilbomycetes*, one isolate was identified only at the order level (K65 B05), one at the family level (C23 B33), and 15 at the species level (Fig. 2). Isolate K65 B05 was placed distantly from other species in the family *Chionosphaeraceae*, leading to its designation as *Agaricostilbales* sp. Isolate C23 B33 was clustered in a clade with reference sequences from the genera *Kurtzmanomyces* and *Mycogloea nipponica*. The closest sequence corresponds to the isolate *Kurtzmanomyces* sp. KBP Y-6546 from the lichen *Cladonia stellaris* in Russia (Kachalkin et al. 2024). However, our isolate was not conspecific with any described species, and since we have only one isolate, it was designated as *Chionosphaeraceae* sp. Finally, 15 isolates formed a well-supported monophyletic clade within the genus *Boekhoutia* yet diverged from the two described species of this genus. The isolates differed from the closely related species *B. foliicola* CGMCC 2.6878 (OP470303/OP470207) by 19 nucleotides and 16 gaps in the LSU region and by four nucleotide mismatches and three gaps in the ITS region. Compared to *B. sterigmata* CGMCC 2.4539 (MK050371), there were 16 nucleotide substitutions and 12 gaps in the ITS region. In addition, there are also strong physiological differences between the species (see diagnosis in the Taxonomy section) (Table S2). Therefore, these isolates were classified as representatives of a new species, which we have named *Boekhoutia peltigerae* sp. nov.

### Class *Cystobasidiomycetes*

**Fig. 3:**
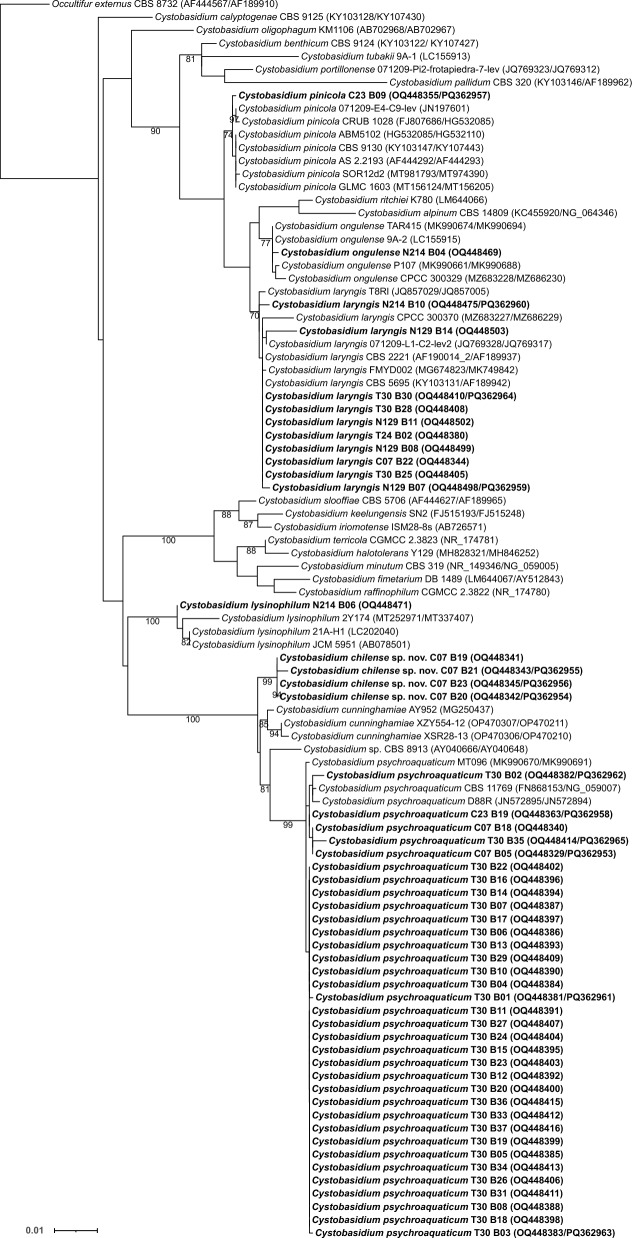
Phylogenetic relationships of yeast isolates obtained from *Peltigera* and related taxa in the genus *Cystobasidium* (*Cystobasidiomycetes*). The dataset included all sequenced species in the genus (Li et al. 2020), except for *C. onofrii*, which was otherwise not closely related to our isolates (Leo et al. 2023). We included an extended sampling in *C. cunninghamiae*, *C. laryngis*, *C. lysinophilum*, *C. ongulense*, *C. pinicola*, and *C. psychroaquaticum*, as the closest relatives to our isolates. *Occultifur externus* was used as outgroup based on Li et al. (2020). The alignment included 95 terminals with 1203 characters—136 of which were parsimony-informative and 963, constant. The substitution model GTR + F + I + G4 was selected for the ITS1, the HKY + F + I for the 5.8S, the GTR + F + I + G4 for the ITS2, and the TIM2 + F + I + G4 for the LSU. We considered four independent partitions, ITS1 (1–205), 5.8S (206–360), ITS2 (361–589) and LSU (590–1203). Maximum likelihood bootstrap values ≥ 70% are indicated below branches. The isolates obtained in this work are highlighted in bold

Within the class *Cystobasidiomycetes*, a total of 51 isolates were identified as members of the genus *Cystobasidium* (Fig. 3). Specifically, isolate N214 B04 was identified as *C. ongulense*, and isolate C23 B09 was identified as *C. pinicola*. Ten isolates were grouped into a well-supported monophyletic clade and were designated as *C. laryngis*, while the other 34 isolates formed a well-supported clade and were designated as *C. psychroaquaticum*. Isolate N214 B06 (GenBank: OQ448471), although it showed three nucleotide substitutions and one gaped position to the type, grouped into a well-supported monophyletic clade with *C. lysinophilum* and was classified as belonging to this species. Four isolates formed a distinct well-supported monophyletic clade, clearly separated from the closest known species, *C. cunninghamiae* and *C. psychroaquaticum*. They differed from the closely related species *C. cunninghamiae* XSR28-13 (OP470306/OP470210) by four nucleotides in the LSU, eight nucleotide mismatches, and one gap in the ITS region. These species exhibit significant physiological differences with our isolates (see diagnosis in the Taxonomy section) (Table S3). The phylogenetic placement, sequence heterogeneity, and differences in physiological profiles from closely related species suggest that these isolates belong to a new species of the *Cystobasidium*, which we named *Cystobasidium chilense* sp. nov.

### Class *Microbotryomycetes*

Within the class *Microbotryomycetes*, two isolates were identified as belonging to the genus *Rhodotorula* (Fig. S3). Isolate C07 B24 clustered within a well-supported clade with sequences of *R. kratochvilovae*. Isolate T30 B38, which grouped with sequences of *R. glutinis*, showed only two and one gaps, respectively, compared to the type CBS 20 (GenBank: AF444539, AF070430) and was thus classified as *R. glutinis*.

### Class *Tremellomycetes*

Within the class *Tremellomycetes*, 109 isolates were classified among the orders *Cystofilobasidiales*, *Filobasidiales*, *Holtermanniales*, and *Tremellales*.

### Order *Cystofilobasidiales*

Within the order *Cystofilobasidiales*, four isolates belonged to the genus *Tausonia* (Fig. S4). These isolates displayed a close relationship to the reference sequences of *T. pullulans*, including the type (strain CBS 2532). The isolates were all designated as *T. pullulans*.

### Order *Filobasidiales*

Within the order *Filobasidiales,* 64 isolates belonged to four genera of basidiomycetes yeasts: *Filobasidium*, *Goffeauzyma*, *Naganishia,* and *Solicoccozyma*.

Within the genus *Filobasidium*, ten isolates were assigned to two distinct clades (Fig. S5). The first clade, comprising five isolates, formed a well-supported monophyletic group closely related to *F. wieringae.* The second clade, consisting of five isolates, was closely related to the sequence of *F. stepposum*.

Within the genus *Goffeauzyma*, five isolates were identified as *G. gastrica*, as they formed a well-supported clade with sequences from this species (Fig. S6).

A total of 22 isolates were placed in the genus *Naganishia* (Fig. S7). The phylogenetic analysis revealed a well-supported clade that included five isolates clustering with the sequences of *N. adeliensis,* and another well-supported clade comprising eight isolates clustered with *N. onofrii*. Both clades showed high sequence similarity to the respective sequences from the type material, CBS 8351 and DBVPG 5303. Three isolates N214 B01, N214 B14, and N214 B18 clustered with sequences of several species. Among them, the sequences of our isolates showed high similarity to sequences of *N. albidosimilis*, *N. diffluens*, and *N. tulchinskyi*, which share identical ITS sequences and can be only distinguished by multi-locus and whole-genome phylogenetic analyses (Bijlani et al. 2022). The cluster additionally included four sequences of *Naganishia* sp. obtained from *Rhizoplaca melanophthalma* lichens collected in Argentina (OP045990-OP045992) and Chile (OP045993) (Cometto et al. 2022). Without additional data, it is not possible to identify these yeasts at a species level. The other six isolates formed a well-supported clade together with three sequences from GenBank, two identified as *N. antarctica* (AY663 and DBVPG 5272) and one—probably misidentified—as *Goffeauzyma gilvescens* (M5-4C-3C). This is a distinct clade sister to the one including sequences from the type strain of *N. antarctica* (CBS 7687). This prevents us from confidently assigning our isolates as *N. antarctica*, but the genetic distances are too small to characterize the clade as a new species. Therefore, they are left unnamed as *Naganishia* sp., for now. *Naganishia* includes several still unresolved species complexes in need of further investigation. Multilocus or even phylogenomic studies will probably be needed to elucidate species boundaries in this genus.

Within the genus *Solicoccozyma*, 27 strains were identified (Fig. S8). A clade of 11 isolates formed a well-supported group with sequences from various isolates of *S. terricola*, including sequence of the type (CBS 4517). A separate clade, composed of 16 isolates, grouped into a well-supported clade with sequences of different strains of *S. gelidoterrea*.

### Order *Holtermanniales*

Within the order *Holtermanniales*, 29 isolates belonging to the genus *Holtermanniella* were found (Fig. S9). Of these, 21 isolates were closely related to *H. wattica*. The remaining isolates formed two well-supported clades: one was composed of two isolates identified as *H. festucosa*, and the other was composed of six isolates identified as *H. takashimae*.

### Order *Tremellales*

A total of 12 isolates were phylogenetically placed in the order *Tremellales*. Among the isolates, one (T20 B04) was closely related to a yeast sequence obtained from *Rhizoplaca melanophthalma* collected in the USA, identified as *Tremellales* sp. L3738 (Cometto et al. 2022). This isolate was distinct from other sequences within the order *Tremellales*, based on the LSU sequence analysis (Fig. S2). BLAST analysis of the ITS region showed that T20 B04 had the highest similarity to sequences from cultures labeled as *Tremellales* sp., which belong to the so-called Yeast Lineage II clade in Cometto et al. (2022). This yeast clade is placed next to the mycoparasite species *Sirobasidium magnum* and is represented by isolates obtained from lichens *R. melanophthalma* from various localities, including the USA, Spain, Argentina, and Chile (Cometto et al. 2022). The taxonomic placement of this clade is complicated, considering the polyphyletic nature of the genus *Sirobasidium* and the lack of sequence data from the type species of the genus (Kachalkin et al. 2019). Therefore, we have designated T20 B04 as *Tremellales* sp.

**Fig. 4:**
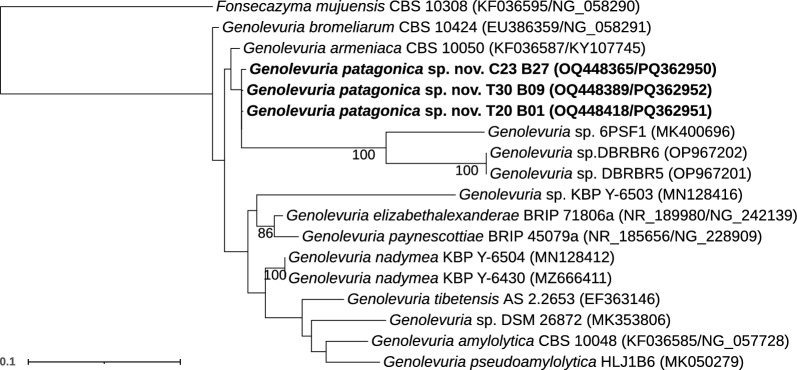
Phylogenetic relationships of yeast isolates obtained from *Peltigera* and related taxa in the genus *Genolevuria* (*Tremellomycetes*). The dataset included sequences of all species accepted in the genus (Liu et al. 2015b; Li et al. 2020; Tan and Shivas 2023; Kachalkin et al. 2024). *Fonsecazyma mujuensis* was used as an outgroup based on Li et al. (2020). The alignment included 18 terminals with 1442 characters—136 of which were parsimony-informative and 1156, constant. The substitution model TNe + FQ + G4 was selected for the ITS1 and the ITS2, and the TNe + FQ + I for the 5.8S and for the LSU. We considered four independent partitions, ITS1 (1–224), 5.8S (225–385), ITS2 (386–653) and LSU (654–1442). Maximum likelihood bootstrap values ≥ 70% are indicated below branches. The isolates obtained in this work are highlighted in bold

Within the genus *Genolevuria*, three isolates were identified (Fig. 4). These isolates were related to *G. armeniaca* CBS 10050 but showed some sequence divergence. Specifically, similarities of 98% (two substitutions and seven gaps) and 97% (nine substitutions and eight gaps) in the LSU and ITS sequences, respectively, suggest our isolates can represent a novel species. Physiologically, our isolates also differ from its close relative *G. armeniaca* (see diagnosis in the Taxonomy section) (Table S4). Based on sequence and physiological differences, our isolates represent a novel species designated *Genolevuria patagonica* sp. nov.

**Fig. 5:**
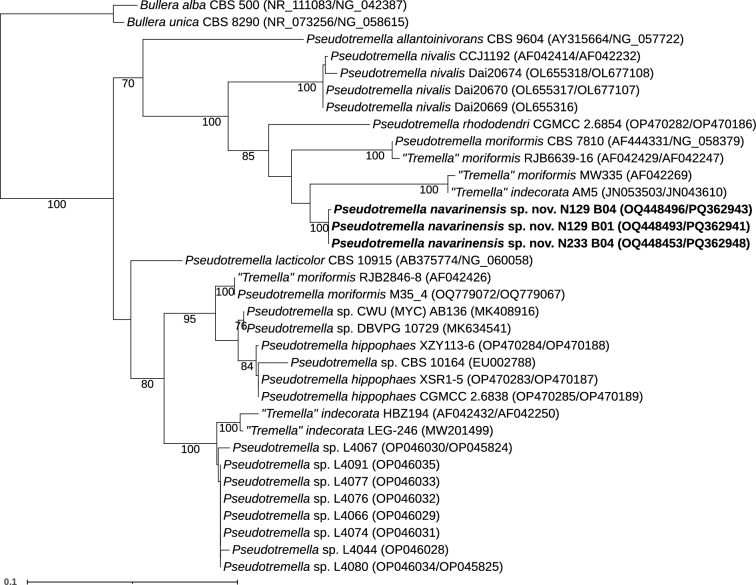
Phylogenetic relationships of yeast isolates obtained from *Peltigera* and related taxa in the genus *Pseudotremella* (*Tremellomycetes*). The dataset included sequences of all species accepted in the genus, and related taxa (i.e., *Tremella indecorata* and *T. moriformis*, Liu et al. (2015a); Li et al. (2020); Jiang et al. (2024). *Bullera alba* and *B. unica were* used as outgroup based on Jiang et al. (2024). The alignment included 34 terminals with 991 characters—198 of which were parsimony-informative and 739, constant. The substitution model TNe + FQ + G4 was selected for the ITS1 and the ITS2, and the TNe + FQ + I + G4 for the 5.8S and for the LSU. We considered four independent partitions, ITS1 (1–91), 5.8S (92–200), ITS2 (201–445) and LSU (446–991). Maximum likelihood bootstrap values ≥ 70% are indicated below branches. The isolates obtained in this work are highlighted in bold

Within the genus *Pseudotremella* (Fig. 5), three isolates formed a well-supported monophyletic clade, distinct from sequences of hitherto described species. These isolates were not closely related to *Pseudotremella* sp. sequences (L4044 to L4091) isolated from *Tephromela atra* lichens (Cometto et al. 2022) or any other isolates from lichens. They differed from the closely related species *Pseudotremella moriformis* CBS 7810 (AF444331/NG_058379) by 27 nucleotides and four gaps in the LSU region, and by 37 nucleotide mismatches and 38 gaps in the ITS region. Compared to the sequence “*Tremella*” *indecorata* AM5 (JN053503/JN043610), there were 31 nucleotides and six gaps in the LSU region, and 33 nucleotide mismatches and 28 gaps in the ITS region. Besides, our isolates differ from the closely related *P. moriformis* and *P. lacticolor* (see diagnosis in the Taxonomy section) (Table S5). Therefore, based on the differences, we propose that our isolates represent a new species, *Pseudotremella navarinensis* sp. nov.

Within *Teunia* (Fig. S10), three isolates exhibited a close relationship to the types *T. betulae* CBS 13896 and *T. turchettiae* KBP Y-6607. However, they showed a close relationship with *T. betulae*, and therefore, they were named as this species.

Within the genus *Vishniacozyma*, two isolates were identified (Fig. S11). Isolate N214 B02 formed a well-supported clade with the sequences labeled as *V. dimennae*, including the type CBS 5770, and was therefore assigned to this species. The other isolate, T20 B13, formed a well-supported clade with sequences of *V. tephrensis,* including the type CBS 8935, and was designated as this species*.*

### Basidiomycete yeasts isolated from *Peltigera* lichens

Out of all the basidiomycete yeasts isolated, 167 were identified at the species level, nine at the genus level, one at the family level, and two isolates were identified only at the order level. *Peltigera* lichens harbored diverse yeasts, with members of the class *Tremellomycetes* comprising 61% of all the isolates and contributed to the overall richness values with 10 genera. Yeasts from the classes *Agaricostilbomycetes*, *Cystobasidiomycetes*, and *Microbotryomycetes* were less common in the studied samples and showed limited taxonomic diversity, primarily including isolates from the family *Chionosphaeraceae*, and the genera *Cystobasidium* and *Rhodotorula,* respectively (Table S1).

Specifically, twenty isolates across nine taxa were obtained from *P. frigida*, while 19 isolates representing eight taxa were found in *P. “fuscopraetextata”*. “*P. ponojensis/monticola* 11” contained 48 isolates spanning 15 taxa, and 92 isolates representing 18 taxa were observed in *P. rufescens*. Notably, *P. rufescens* exhibited the highest richness of yeasts. The genus *Cystobasidium* had the highest occurrence among all isolates, followed by *Holtermanniella*, *Solicoccozyma*, and *Naganishia. H. wattica* and *S. terricola* were found in all four *Peltigera* species (Table 2).

**Table 2:** Species of basidiomycete yeasts isolated from *Peltigera* lichens and their abundance

Species	*Peltigera frigida*	*Peltigera* “*fuscopraetextata*”	“*Peltigera ponojensis/monticola* 11”	*Peltigera rufescens*
*Agaricostilbomycetes*				
*Agaricostilbales* sp.	–	1	–	–
*Boekhoutia peltigerae* sp. nov.	1	1	–	13
*Chionosphaeraceae* sp.	–	–	–	1
*Cystobasidiomycetes*				
*Cystobasidium chilense* sp. nov.	–	–	4	–
*Cystobasidium laryngis*	–	4	2	4
*Cystobasidium lysinophilum*	–	–	–	1
*Cystobasidium ongulense*	–	–	–	1
*Cystobasidium pinicola*	–	–	–	1
*Cystobasidium psychroaquaticum*	–	–	2	32
*Microbotryomycetes*				
*Rhodotorula glutinis*	–	–	–	1
*Rhodotorula kratochvilovae*	–	–	1	–
*Tremellomycetes*				
*Filobasidium stepposum*	–	1	–	4
*Filobasidium wieringae*	–	–	2	3
*Genolevuria patagonica* sp. nov.	1	–	–	2
*Goffeauzyma gastrica*	–	–	3	2
*Holtermanniella festucosa*	–	–	2	–
*Holtermanniella takashimae*	–	–	6	–
*Holtermanniella wattica*	4	4	7	6
*Naganishia adeliensis*	–	–	3	2
*Naganishia onofrii*	1	–	7	–
*Naganishia* sp.	–	–	6	3
*Pseudotremella navarinensis* sp. nov.	–	2	1	–
*Solicoccozyma gelidoterrea*	2	–	1	13
*Solicoccozyma terricola*	5	3	1	2
*Tausonia pullulans*	4	–	–	–
*Teunia betulae*	–	3	–	–
*Tremellales* sp.	1	–	–	–
*Vishniacozyma dimennae*	–	–	–	1
*Vishniacozyma tepherensis*	1	–	–	–

Identification of already described taxa was based on a criterion of monophyly and pair-wise comparisons of genetic distances within and between species. Four new species of basidiomycete yeasts associated with *Peltigera* lichens from southern Chile were described based on a combination of phylogenetic and physiological data: *Boekhoutia peltigerae* sp. nov., *Cystobasidium chilense* sp. nov., *Genolevuria patagonica* sp. nov., and *Pseudotremella navarinensis* sp. nov.

### Taxonomy

#### *Boekhoutia peltigerae* Y. Pérez, Yurkov, Serey & Orlando, sp. nov.

**Mycobank No:** MB856739

**Etymology:** The specific epithet *peltigerae* refers to *Peltigera*, the host lichen genus from which isolates of this species were isolated.

**Diagnosis:** Physiologically, *B. peltigerae* differs from the closely related *B. sterigmata* (CGMCC 2.4539) by the utilization of d-arabinose, d-mannitol, glycerol, l-rhamnose, d-raffinose, potassium nitrate and differs from *B. foliicola* (CGMCC 2.6878) in the utilization of d-arabinose, l-arabinose, d-xylose, ethanol, glycerol, l-rhamnose and sodium nitrate (Table S2). It also differs in the host, which in this case is lichens.

**Type: Holotype:** Chile: Coyhaique National Reserve, Coyhaique province, Aysén Region, 45° 32′ 10.3″ S, 72°1′ 14.2″ W, from the thallus of the lichen *Peltigera rufescens*, July 2022, *Orlando J* [isol. Pérez Y and Serey N]. The holotype is preserved in a metabolically inactive state in the Chilean Collection of Microbial Genetic Resources (CChRGM, RGM3670). The ex-type culture is maintained in the Microbial Ecology Laboratory at the Universidad de Chile (C23 B28). GenBank accessions ITS–OQ448366, LSU-PQ362979.

**Description:** On Yeast Malt Agar (YMA), after 5 days at 15 °C, the colony appears orange to pink, shiny, mucoid, and viscous, with a smooth, entire margin. The colonies are circular, measuring 0.5 × 0.5 mm. Cells are oval (5 × 7.5 μm), with budding observed. Germ tubes are visible, and no mycelial structures were detected. Physiological and biochemical characteristics: Glucose fermentation is absent. d-arabinose (delayed), d-gluconate (delayed), d-mannitol (weak), d-galactose, d-glucose, glycerol, l-rhamnose, maltose, d-raffinose, sucrose, cellobiose (delayed) are assimilated as sole carbon sources. Sodium citrate, l-arabinose, d-xylose, i-erythritol, ethanol, lactose, melibiose, methanol, and *N*-acetyl-d-glucosamine are not assimilated. Ammonium sulfate, creatinine, and potassium nitrate are assimilated as sole nitrogen sources, and sodium nitrite is not assimilated. No growth on 10% NaCl and 5% glucose medium. Starch-like compounds are not produced. Growth temperature between 4 and 15 °C. No growth at 30 °C.

**Notes:** *Boekhoutia peltigerae* differed from the closely related species *B. foliicola* CGMCC 2.6878 (OP470303/OP470207) by 19 nucleotides and 16 gaps in the LSU region and by four nucleotide mismatches and three gaps in the ITS region. Compared to *B. sterigmata* CGMCC 2.4539 (MK050371), it showed 16 nucleotide substitutions and 12 gaps in the ITS region.

**Substrate/Host:** Thalli of lichens *Peltigera rufescens*, *P*. “*fuscopraetextata*” and *P. frigida.*

**Distribution:** Chile.

#### *Cystobasidium chilense* Y. Pérez, Yurkov, Serey & Orlando, sp. nov.

**Mycobank No:** MB856738

**Etymology:** The specific epithet *chilense* refers to Chile, the country where the *Peltigera* lichen, from which the yeasts were isolated, was collected.

**Diagnosis:** Physiologically, *C. chilense* differs from the closely related *C. cunninghamiae* (CGMCC 2.6841) by utilization of l-arabinose, i-erythritol, d-galactose, l-rhamnose, maltose, melibiose, cellobiose, d-glucosamine, d-ribose, d-sorbitol, d-raffinose and α-methyl-d-glucoside, sodium nitrite, differs from *C. psychroaquaticum* (K-833) by the utilization of d-arabinose, i-erythritol, d-galactose, maltose, melibiose, cellobiose, d-ribose, l-sorbose, α-methyl-d-glucoside, creatine and potassium nitrate, and differs from *C. lysinophilum* (JCM 5951) by the utilization of i-erythritol, lactose, d-glucosamine, salicin, l-sorbose, potassium nitrate and growth at 30 °C (Table S3).

**Type: Holotype:** Chile, Coyhaique National Reserve, Coyhaique province, Aysén Region, 45° 32′ 10.7″ S, 72° 1′ 11.6″ W, from the thallus of the lichen “*Peltigera ponojensis/monticola* 11”, July 2022, *Orlando J* [isol. Pérez Y and Serey N]. The holotype is preserved in a metabolically inactive state in the Chilean Collection of Microbial Genetic Resources (CChRGM, RGM 3661). The ex-type culture is maintained in the Microbial Ecology Laboratory at the Universidad de Chile (C07 B20). GenBank accessions ITS-OQ448342 and LSU-PQ362954.

**Description:** On Yeast Malt Agar, after 5 days at 15 °C, the streak culture appears salmon-orange to pinkish, shiny, mucilaginous, smooth, with smooth entire margins. Cells are ovoid (5 x 7.5 μm), with polar budding observed. Ballistoconidia, pseudohyphae, and mycelial structures were not detected. Physiological and biochemical characteristics: Glucose fermentation is absent. d-arabinose (weak), l-arabinose, d-mannitol, d-xylose, i-erythritol (variable), d-galactose (variable), d-glucose, glycerol, l-rhamnose (variable), maltose (weak), maltriose, melibiose (weak), melezitose, sucrose, cellobiose, d-glucosamine (variable), *N*-acetyl-d-glucosamine, d-ribose (weak), d-sorbitol, adonitol, d-arabitol, inulin, d-raffinose (weak), salicin, l-sorbose, fumaric acid, l-malic acid, succinic acid monomethyl ester (variable), bromosuccinic acid, l-glutamic acid, γ-aminobutyric acid, α-ketoglutaric acid, 2-keto-gluconic acid, d-gluconic acid, dextrin, gentibiose, palatinose (weak), stachyose (weak), d-trehalose, turanose, d-psicose, α-methyl-d-glucoside (variable), β-methyl-d-glucoside, amygdalin, arbutin, salicin, maltitol, xylitol, methyl succinate + d-xylose, *N*-acetyl-l-glutamic acid + d-xylose, quinolic acid + d-xylose, d-glucuronic acid + d-xylose, dextrin + d-xylose, d-melibiose + d-xylose, d-galactose + d-xylose, m-inositol + d-xylose, 1,2-propanediol + d-xylose, acetoin + d-xylose are assimilated as sole or combination of carbon sources. Sodium citrate, citric acid, myo-inositol, and lactose are not assimilated. Ammonium sulfate, creatine (weak), and potassium nitrate are assimilated as sole nitrogen sources, while sodium nitrite is not. There is no synthesis of extracellular amyloid compounds. Growth temperature between 4 and 25 °C. No growth at 30 °C.

**Notes:** *Cystobasidium chilense* differed from the closely related species *C. cunninghamiae* XSR28-13 (OP470306/OP470210) by four nucleotides in the LSU region, eight nucleotide mismatches, and one gap in the ITS region.

**Substrate/Host:** Thallus of lichen “*Peltigera ponojensis/monticola* 11”.

**Distribution:** Chile.

#### *Genolevuria patagonica* Y. Pérez, Yurkov, Serey & Orlando, sp. nov.

**Mycobank No:** MB856740

**Etymology:** The specific epithet *patagonica* refers to Chilean Patagonia, the geographical region where the *Peltigera* lichen, from which the yeasts were isolated, was collected.

**Diagnosis:** Physiologically, *Genolevuria patagonica* differs from its close relative *G. armeniaca* (CBS 10050) by the utilization of sodium citrate, d-mannitol, ethanol, creatine, creatinine, potassium nitrate, and the synthesis of extracellular amyloids; and differs from *G. bromeliarum* (CBS 10424) by the utilization of d-mannitol, ethanol, glycerol, cellobiose, creatine, creatinine and sodium nitrite (Table S4).

**Type: Holotype:** Chile: Patagonia National Park, Tamango area, Aysén Region, from the thallus of the lichen *Peltigera frigida*, July 2022, *Orlando J* [isol. Pérez Y and Serey N]. The holotype is preserved in a metabolically inactive state in the Chilean Collection of Microbial Genetic Resources (CChRGM, RGM 3631).  The ex-type culture is maintained in the Microbial Ecology Laboratory at the Universidad de Chile (T20 B01). GenBank ITS-OQ448418 and LSU-PQ362951.

**Description:** On Yeast Malt Agar (YMA), after 5 days at 15 °C, the colony appears light orange, shiny and viscous, with a smooth, entire margin. The colonies are circular, measuring 0.2 × 0.2 mm. Cells are oval (2.5 × 7.5 μm), and exhibited polar budding. No mycelial structures were detected. Physiological and biochemical characteristics: Glucose fermentation is absent. d-arabinose, l-arabinose, d-gluconate (delayed), d-mannitol (variable), d-xylose, ethanol (variable), d-galactose, d-glucose, glycerol (delayed), meso-inositol, lactose, l-rhamnose, maltose, melibiose, *N*-acetyl-d-glucosamine (delayed), d-raffinose (delayed), sucrose, cellobiose are assimilated as sole carbon sources. Sodium citrate, i-erythritol, and methanol are not assimilated. Ammonium sulfate (delayed), creatine, creatinine, and potassium nitrate are assimilated as sole nitrogen sources, while sodium nitrite is not. There is no growth in vitamin-free medium, 10% NaCl and 5% glucose medium, or 50% glucose-yeast extract Agar. Growth temperature between 4 and 25 °C. No growth at 30 °C.

**Substrate/Host:** Thalli of lichens *Peltigera rufescens* and *Peltigera frigida.*

**Notes:** *Genolevuria patagonica* differed from the closely related species *G. armeniaca* CBS 10050 (KF036587/KY107745) by two nucleotide substitutions and seven gaps in the LSU, and by nine nucleotide substitutions, and eight gaps in the ITS region.

**Distribution:** Chile.

#### *Pseudotremella navarinensis* Y. Pérez, Yurkov, Serey & Orlando, sp. nov.

**Mycobank No:** MB856724

**Etymology:** The specific epithet *navarinensis* refers to Isla Navarino in Chile, the location where the *Peltigera* lichen, from which the yeasts were isolated, was collected.

**Diagnosis:** Physiologically, *P. navarinensis* differs from the closely related *P. moriformis* (CBS 7810) by the utilization of i-erythritol, ethanol, melibiose and sodium nitrite assimilation, growth on vitamin-free medium, growth on 50% glucose-yeast extract agar, synthesis of extracellular amyloids and 30 °C grow temperature, differs from *P. allantoinivorans* (CBS 9604) by the utilization of its potassium nitrate, synthesis of extracellular amyloids and growth at 30 °C, and differs from *P. lacticolor* (CBS 10915) by the utilization of potassium nitrate, synthesis of extracellular amyloids, growth on 50% glucose-yeast extract agar and growth at 30 °C. *P. navarinensis* generally differs from other described species of *Pseudotremella* (Table S5).

**Type: Holotype:** Chile: Navarino Island, Cape Horn, Magallanes and Chilean Antarctica Region, 54° 57′ 17.6″ S 67° 37′ 58.2″ W, from the thallus of the lichen *Peltigera “fuscopraetextata”*, May 2022, *Orlando J* [isol. Pérez Y and Serey N]. The holotype is preserved in a metabolically inactive state in the Chilean Collection of Microbial Genetic Resources (CChRGM, RGM 3659). The ex-type culture is maintained in the Microbial Ecology Laboratory at the Universidad de Chile (N129 B01). GenBank ITS-OQ448493 and LSU-PQ362941.

**Description:** On Yeast Malt Agar (YMA), after 5 days at 15 °C, the colony appears cream, shiny, mucoid and viscous, with a smooth, entire margin. The colonies are circular, measuring 1 × 1 mm. Cells are oval (2.5 × 7.5 μm), and exhibited polar budding. No mycelial structures were detected. Physiological and biochemical characteristics: Glucose fermentation is absent. Sodium citrate (delayed), d-arabinose, l-arabinose, d-gluconate, d-mannitol, d-xylose, i-erythritol, ethanol, d-galactose, d-glucose, glycerol, meso-inositol, lactose, l-rhamnose, maltose, melibiose, *N*-acetyl-d-glucosamine, d-raffinose (delayed), sucrose, cellobiose are assimilated as sole carbon sources. Methanol is not assimilated. Ammonium sulfate (delayed), creatinine, creatine and potassium nitrate are assimilated as sole nitrogen sources and sodium nitrite is not assimilated. Growth on vitamin-free medium, weak growth on 10% NaCl and 5% glucose medium, growth on 50% glucose-yeast extract agar. Extracellular amyloids are not produced. Growth temperature between 4 and 25 °C. No growth at 30 °C.

**Notes:** *Pseudotremella navarinensis* differed from species *Pseudotremella moriformis* CBS 7810 (AF444331/NG_058379) by 27 nucleotides and four gaps in the LSU, and by 37 nucleotide mismatches and 38 gaps in the ITS region. Compared to the sequence “*Tremella*” *indecorata* AM5 (JN053503/JN043610), there were 31 nucleotides and six gaps in the LSU, and 33 nucleotide mismatches and 28 gaps in the ITS region.

**Substrate/Host:** Thalli of lichens *Peltigera “fuscopraetextata”* and *“Peltigera ponojensis/monticola* 11”.

**Distribution:** Chile.

## Discussion

Lichen thalli host a wide array of fungal communities, including basidiomycete yeasts (Muggia and Grube 2018; Kachalkin et al. 2024). In recent years, these yeasts have gained significant attention as research efforts focus on unraveling their diversity and their roles within the lichen symbiosis (Spribille et al. 2016; Černajová and Škaloud 2019; Tuovinen et al. 2019, 2021; Lendemer et al. 2019; Mark et al. 2020; Smith et al. 2020; Tagirdzhanova et al. 2021). These investigations have predominantly employed molecular techniques, with culture-dependent methods playing a relatively minor role. The limited use of culture-dependent techniques may be attributed to the challenges associated with cultivating basidiomycete yeasts, such as the prevalent inability to culture members of the order *Cyphobasidiales* despite their widespread occurrence in lichens (Spribille et al. 2016). However, there has been a recent increase in culture-dependent studies, employing various strategies to successfully isolate and culture yeasts associated with the thalli of different lichen species (Duarte et al. 2013, 2016; Santiago et al. 2015; Cometto et al. 2022; da Silva et al. 2022b; Raudabaugh and Aime 2023; Kachalkin et al. 2024). These efforts have led to the discovery of novel species of basidiomycete yeasts from previously undescribed lichens (Černajová and Škaloud 2019; Nguyen et al. 2023). In the present study, the isolation and identification of four new species of basidiomycete yeasts show the importance of traditional cultivation techniques in discovering and characterizing yeasts from lichens. Cultivation-based methods remain essential in microbiology, especially when exploring understudied microbial habitats, where methods like metagenomics might otherwise overlook fungi present in low abundance. Viable cultures enable detailed physiological and molecular analyses for a more comprehensive understanding of microbial ecosystems.

Basidiomycete yeasts have been documented in association with various lichen species across vast geographical distances and on six continents, but the basidiomycetes inhabiting *Peltigera* lichens remain poorly characterized. To our knowledge, only one study has successfully isolated basidiomycete yeasts from *P. membranacea* in Russia (Kachalkin et al. 2017). Here, 179 isolates of basidiomycete yeasts were recovered from the thalli of four *Peltigera* species collected from different sampling sites, both grassland and forest habitats in southern Chile. The basidiomycete yeasts were isolated from all samples except for the *P.* “*fuscopraetextata*” specimen collected in 2013 in Coyhaique, possibly due to the nine-year time lapse between thallus collection and cultivation of the yeasts. Yeast isolation from lichens is commonly done from freshly collected samples. However, the successful cultivation of yeasts from lichen samples collected in 2018 highlights the remarkable resilience of these microorganisms. The recovery of yeasts from lichen thalli suggests the broad ecological importance of lichens as hosts and habitats for diverse microorganisms.

The yeast isolates obtained in this study add to previous studies where basidiomycete yeasts were isolated from other lichen species, which also reported a higher number of isolated basidiomycete yeasts within *Tremellomycetes* (Cometto et al. 2022; Raudabaugh and Aime 2023; Kachalkin et al. 2024). While yeasts belonging to the class *Cystobasidiomycetes* have been detected in numerous lichen species, these findings have primarily been based on molecular techniques utilizing specific primers for this class (Spribille et al. 2016; Černajová and Škaloud 2019; Mark et al. 2020). In contrast, culture-dependent studies have demonstrated a relatively lower success rate in isolating representatives of the class *Cystobasidiomycetes* compared to other classes of basidiomycetes (Cometto et al. 2022; Raudabaugh and Aime 2023), though the trend is not fully universal as demonstrated for *Cladonia* lichens (Kachalkin et al. 2024).

Some species of basidiomycete yeast associated with *Peltigera* in this study have been previously reported in other lichen species. For example, *C. pinicola, C. psychroaquaticum,* and *S. terricola* have been previously isolated from *Cladonia* lichens (Zhang et al. 2016; Černajová and Škaloud 2020; Kachalkin et al. 2024), while *C. laryngis* and *T. pullulans* have been previously isolated from Antarctic lichen species (Duarte et al. 2013, 2016; Santiago et al. 2015; da Silva et al. 2022b). Moreover, *F. wieringae* has been isolated from *Rhizoplaca melanophthalma* and *Tephromela atra* (Cometto et al. 2024), and *H. wattica* and *N. adeliensis* have been associated with *Ramalina terebrata* (Duarte et al. 2016) and *Usnea antarctica* (Santiago et al. 2015; Duarte et al. 2016; Pankratov et al. 2017). At the same time, the aforementioned yeasts have been isolated from other substrates and environments not linked to lichens. The discovery of these yeasts deep in lichen tissues and not on the surface suggests lichens can provide a living room to yeasts (Kachalkin et al. 2024). Similarly, our study yielded yeasts that belong to the novel yet unplaced lineages in the order *Agaricostilbales* and the family *Chionosphaeraceae*. Previous studies have reported the isolation of representatives of the family *Chionosphaeraceae* from *R. melanophthalma* and *T. atra* lichens collected in the USA and Italy (Cometto et al. 2022) and *Cladonia* collected in Russia (Kachalkin et al. 2024). Even though these isolates differ from yeasts obtained in the present study, we can state that *Peltigera* lichens also harbor these rare yeasts.

This study also documents several yeast species in lichens for the first time, expanding our understanding of lichen-associated fungal diversity. This includes *C. ongulense, F. stepposum, G. gastrica, H. festucosa, H. takashimae, N. onofrii, R. kratochvilovae, R. glutinis, S. gelidoterrea, T. betulae, V. tephrensis*, and *V. dimennae.* Although most of these genera have previously been identified in lichens, this study marks the first documentation of specific species within lichens. Moreover, the genus *Goffeauzyma* is documented in lichens here for the first time.

Some of these yeasts, such as *C. ongulense*, *G. gastrica*, *N. onofrii*, *R. glutinis*, *S. gelidoterrea*, *V. tephrensis*, and *V. dimennae* are found in soils (Fell and Phaff 1967; Vishniac 2002; Turchetti et al. 2015; Tsuji et al. 2017; Li et al. 2020; Nutaratat et al. 2022; He et al. 2024). *Peltigera* lichens have shown the capacity to acquire key microbial symbionts directly from the soils in which they grow (Zúñiga et al. 2017; Leiva et al. 2021), suggesting that their local soil environment serves as a reservoir for vital components of their microbiome. These yeasts could be transferred to lichen thalli directly from soil particles or through fungal spores that inhabit the soil. Another potential source of yeasts is plant material, with several prominent phylloplane yeasts as *F. stepposum*, *R. kratochvilovae*, *H. festucosa*, *H. takashimae*, and *T. betulae* (Golubev et al. 2004, 2006; Wuczkowski et al. 2011; Jiru et al. 2017; Wang et al. 2020). The potential dual sourcing from both soil and phylloplane environments highlights the adaptive strategies of *Peltigera* lichens in acquiring beneficial microorganisms, thus enhancing their ecological resilience and supporting their role in ecosystem nutrient dynamics.

Most yeast species associated with *Peltigera* lichens have previously predominantly been reported from temperate to cold environments, indicating their psychrotolerant characteristics (de Garcia et al. 2012; Zhou et al. 2020; Guo et al. 2022). These yeasts present valuable adaptations to colonize habitats and survive under harsh environmental conditions, including desiccation, UV stress, and scarce nutrients (Buzzini et al. 2018). Their adaptation to cold conditions allows for continued metabolic activity even in harsh temperatures, contributing to the lichen’s overall survival and ecological functionality. This specialization for colder environments likely gives *Peltigera* lichens a unique advantage in occupying niches with fewer competitors, facilitating their growth in soils and on surfaces that other microbial communities may not easily colonize. Therefore, the psychrotolerant nature of these yeasts not only supports the adaptability and resilience of *Peltigera* lichens but also underscores their role in sustaining ecosystem processes in temperate to cold environments.

Moreover, this study reports the discovery of four new species of basidiomycete yeasts: *Boekhoutia peltigerae* sp. nov., *Cystobasidium chilense* sp. nov., *Genolevuria patagonica* sp. nov*.* and *Pseudotremella navarinensis* sp. nov*. C. chilense* is a newly identified species within the genus *Cystobasidium*, a group already recognized for its ecological diversity and adaptation to a wide variety of habitats. Species in the *Cystobasidium* genus inhabit a broad range of environments, including aquatic ecosystems, soil, sediments, plant surfaces, lichens, and even human-related samples and food products (Fotedar et al. 2019; Černajová and Škaloud 2020; Bazhenova et al. 2021; Karajacob et al. 2022). Adding *C. chilense* to the genus and the first report of *C. ongulense* in lichens improves the growing evidence that *Cystobasidium* species are well-adapted to lichen habitats. *G. patagonica* represents, to our knowledge, the second documented species within the *Genolevuria* genus that forms associations with lichens. Earlier, Raudabaugh and Aime (2023) isolated unidentified *Genolevuria* species from various lichen types, and *Genolevuria nadyme*a was documented in association with the lichen *Cladonia stellaris* (Kachalkin et al. 2024), marking the first known occurrences of this genus interacting with lichen species. *P. navarinensis* is a new species of the genus *Pseudotremella*, a genus reported in lichens as *T. atra* (Cometto et al. 2022) and *Candelaria concolor* (Raudabaugh and Aime 2023), although no specific species had been recorded. *B. peltigerae* represents the third known species of *Boekhoutia.* While the other two species were found on plant leaves in China (Li et al. 2020; Jiang et al. 2024), *B. peltigerae* is the first *Boekhoutia* species isolated from lichens. These findings are significant as they expand the known ecological range of the *Genolevuria*, *Pseudotremella*, and *Boekhoutia* genera, which have primarily been associated with plants, indicating an ecological expansion.

Southern Chile is a highly diverse region that remains poorly studied, specifically in lichenological and microbiological terms (Leiva et al. 2016). Despite limited research, available studies indicated a significant portion of species in this area are endemic, signifying a crucial reservoir of biodiversity (Arroyo et al. 1996). The conservation stage of the habitats influences the diversity of *Peltigera* lichens growing in them *(i.e.*, undisturbed environments have a higher diversity of *Peltigera* lichens), which in turn affect the lichen-associated microbial communities (Ramírez-Fernández et al. 2013, 2014). The presence of numerous basidiomycete yeasts, encompassing new taxa, linked to *Peltigera* lichens in southern Chile further supports the importance of these lichens as an important source of biodiversity. Thus, *Peltigera* lichens contribute to the understanding of local ecosystems but also stand out as potential reservoirs of undiscovered microbial diversity.

## Conclusion

Employing a culture-dependent approach, basidiomycete yeasts were successfully isolated from *P. frigida*, *P. “fuscopraetextata”*, “*P. ponojensis/monticola* 11”, and *P. rufescens* lichens collected across four sites in southern Chile. Based on ITS and LSU sequencing, the phylogenetic analyses revealed diverse yeast communities, encompassing representatives from four classes and 13 genera. Complementing the phylogenetic analyses with physiological studies led to the identification for the first time of four new species of basidiomycete yeasts from the thallus of these lichens: *Boekhoutia peltigerae* sp. nov., *Cystobasidium chilense* sp. nov., *Genolevuria patagonica* sp. nov., and *Pseudotremella navarinensis* sp. nov. Notably, the yeasts identified in this study were previously unreported in *Peltigera* lichens, with a substantial portion never isolated from lichens before. A few novel yeasts received a loose phylogenetic placement, suggesting the potential existence of new yeast lineages at the taxonomic level of genus and family that require further formal descriptions. This study aligns with prior research that underscores lichens as fascinating reservoirs of uncharted yeast diversity, offering valuable insights into the complex and previously undiscovered basidiomycete yeasts within the symbiotic relationship with *Peltigera* lichens, encouraging to consider the importance of preserving these lichens to conserve this noteworthy source of biodiversity.

## Supplementary Information

Additional file1 **Table S1** Basidiomycete yeast isolated from *Peltigera* lichens

Additional file2 **Tables S2-S5** Physiological characteristics of the new species described in this study

Additional file3 **Fig. S1** Phylogenetic relationships of yeast isolates obtained from *Peltigera* and related taxa in the subphylum *Pucciniomycotina*. The dataset was based on recently published phylogenetic trees (Jiang et al. 2024; Kachalkin et al. 2024) and included LSU sequences from our isolates from *Peltigera* and sequences representing the 11 accepted orders and four accepted classes in the group (Wang et al. 2015a, b). Two members of the *Ustilaginomycotina*, viz. *Pseudomicrostroma phylloplanum*and *Mycosarcoma (=Ustilago) maydis* were used as outgroups based on Wang et al. (2015b). The alignment included 506 sequences with 680 characters—431 of which were parsimony-informative and 174, constant. We considered only one partition corresponding to the LSU. The substitution model GTR + F + I + Γ4 was selected. Maximum likelihood bootstrap values ≥70% are indicated below branches. The isolates obtained in this work are highlighted in bold.

Additional file4 **Fig. S2** Phylogenetic relationships of yeast isolates obtained from *Peltigera* and related taxa in the class *Tremellomycetes*. The dataset included LSU sequences from our isolates from *Peltigera*, and sequences of most genera representing the 17 families of the five accepted orders in the class (Liu et al. 2015a, b). *Sterigmatomyces halophilus (Agaricostilbomycetes, Pucciniomycotina) *was used as an outgroup based on Liu et al. (2015b). The alignment included 667 sequences with 654 characters—354 of which were parsimony-informative and 205, constant. We considered only one partition corresponding to the LSU. The substitution model SYM + I + Γ4 was selected. Maximum likelihood bootstrap values ≥ 70% are indicated below branches. The isolates obtained in this work are highlighted in bold.

Additional file5 **Fig. S3** Phylogenetic relationships of yeast isolates obtained from *Peltigera* and related taxa in the genus *Rhodotorula (Pucciniomycotina)*. The dataset included a fair representation of the species accepted in the genus (Wang et al. 2015b; Jiang et al. 2024), with an increased sampling in *R. araucariae*, *R. babjevae*, *R. diobovata*, *R. evergladensis*, *R. glutinis*, *R. graminis*, and *R. kratochvilovae*, as the closest relatives to our isolates. *Rhodosporidiobolus nylandii* was used as an outgroup based on Wang et al. (2015a). The alignment included 37 terminals with 1208 characters—150 of which were parsimony-informative and 972, constant. The substitution model K3Pu + F + G4 was selected for the ITS1 and the ITS2, and the TPM3 + FQ + I for the 5.8S and for the LSU. We considered four independent partitions: ITS1 (1-198), 5.8S (199-358), ITS2 (359-574), and LSU (575-1208). Maximum likelihood bootstrap values ≥ 70% are indicated below branches. The isolates obtained in this work are highlighted in bold.

Additional file6 **Fig. S4** Phylogenetic relationships of yeast isolates obtained from *Peltigera* and related taxa in the genus *Tausonia (Tremellomycetes)*. The dataset included sequences of all species accepted in the genus, with an increased sampling in *T. pullulans* as the closest relative to our isolates. *Itersonilia pannonica* was used as an outgroup based on Kachalkin et al. (2019). The alignment included 14 terminals with 639 characters—54 of which were parsimony-informative and 498, constant. The substitution model TIMe + FQ + G4 was selected for ITS1 and ITS2, and the JC for the 5.8S. We considered three independent partitions, ITS1 (1-179), 5.8S (180-354), and ITS2 (355-639). Maximum likelihood bootstrap values ≥ 70% are indicated below branches. The isolates obtained in this work are highlighted in bold.

Additional file7 **Fig. S5** Phylogenetic relationships of yeast isolates obtained from *Peltigera* and related taxa in the genus *Filobasidium (Tremellomycetes)*. The dataset included all sequenced species in the genus (Li et al. 2020), except for *F. chaidanense*, which was otherwise not closely related to our isolates (Wei et al. 2022). *Naganishia adeliensis* was used as an outgroup based on Li et al. (2020). The alignment included 36 terminals with 607 characters—142 of which were parsimony-informative and 399, constant. The substitution model HKY + F + G4 was selected for ITS1 and ITS2, and the JC for the 5.8S. We considered three independent partitions, ITS1 (1-182), 5.8S (183-330), and ITS2 (331-607). Maximum likelihood bootstrap values ≥ 70% are indicated below branches. The isolates obtained in this work are highlighted in bold.

Additional file8 **Fig. S6** Phylogenetic relationships of yeast isolates obtained from *Peltigera* and related taxa in the genus *Goffeauzyma (Tremellomycetes)*. The dataset included sequences of all species accepted in the genus (Liu et al. 2015b; Nutaratat et al. 2022), with an increased sampling in *G. gastrica* and *G. gilvescens*, as the closest relatives to our isolates. *Filobasidium oeirense* was used as an outgroup based on Liu et al. (2015b). The alignment included 20 terminals with 1230 characters—211 of which were parsimony-informative and 915, constant. The substitution model TIM2 + F + G4 was selected for the ITS1 and the ITS2, and the K2P + FQ + I for the 5.8S and the LSU. We considered four independent partitions: ITS1 (1-224), 5.8S (225-385), ITS2 (386-653), and LSU (654-1230). Maximum likelihood bootstrap values ≥ 70% are indicated below branches. The isolates obtained in this work are highlighted in bold.

Additional file9 **Fig. S7** Phylogenetic relationships of yeast isolates obtained from *Peltigera* and related taxa in the genus *Naganishia (Tremellomycetes)*. The dataset included all sequenced species in the genus (Liu et al. 2015b; Bijlani et al. 2022; Leo et al. 2023), except for *Naganishia indica*, which was otherwise not related to our isolates (Crous et al. 2019). *Filobasidium oeirense* was used as an outgroup based on Liu et al. (2015b). The alignment included 80 terminals with 1354 characters—141 of which were parsimony-informative and 1099, constant. The substitution model K3P + FQ + I was selected for the ITS1, the F81 + F for the 5.8S, and the TIM2 + F + I + G4 for the ITS2 and the LSU. We considered four independent partitions: ITS1 (1-169), 5.8S (170-321), ITS2 (322-626), and LSU (627-1354). Maximum likelihood bootstrap values ≥ 70% are indicated below branches. The isolates obtained in this work are highlighted in bold.

Additional file10 **Fig. S8** Phylogenetic relationships of yeast isolates obtained from *Peltigera* and related taxa in the genus *Solicoccozyma (Tremellomycetes)*. The dataset included sequences of all species accepted in the genus (Liu et al. 2015b, Yurkov and Kurtzman 2019; Li et al. 2020), with an increased sampling in *S. aeria*, *S. fuscescens*, *S. gelidoterrea*, *S. phenolica*, *S. terrea*, *S. terricola*, as the closest relatives to our isolates. *Piskurozyma capsuligena* was used as an outgroup based on Li et al. (2020). The alignment included 58 terminals with 661 characters—135 of which were parsimony-informative and 464, constant. The substitution model HKY + F + G4 was selected for ITS1 and ITS2, and the JC for the 5.8S. We considered three independent partitions, ITS1 (1-215), 5.8S (216-382), and ITS2 (383-661). Maximum likelihood bootstrap values ≥ 70% are indicated below branches. The isolates obtained in this work are highlighted in bold.

Additional file11 ** Fig. S9** Phylogenetic relationships of yeast isolates obtained from *Peltigera* and related taxa in the genus *Holtermanniales (Tremellomycetes)*. The dataset included sequences of *Holtermannia* and *Holtermanniella*, the two accepted genera in the order (Wuczkowski et al. 2011; Liu et al. 2015b; Jiang et al. 2024), with an extended sampling in *Holtermaniella festucosa*, *H. takashimae* and *H. wattica*, as the closest relatives to our isolates. *Filobasidium globisporum* was used as an outgroup based on Liu et al. (2015b). The alignment included 61 terminals with 1153 characters—83 of which were parsimony-informative and 934, constant. The substitution model TIM2e + FQ + I + G4 was selected for all partitions. We considered four independent partitions: ITS1 (1-143), 5.8S (144-313), ITS2 (314-545), and LSU (546-1153). Maximum likelihood bootstrap values ≥ 70% are indicated below branches. The isolates obtained in this work are highlighted in bold.

Additional file12 **Fig. S10** Phylogenetic relationships of yeast isolates obtained from *Peltigera* and related taxa in the genus *Teunia (Tremellomycetes)*. The dataset included sequences of all species accepted in the genus (Li et al. 2020; Khunnamwong et al. 2020; Crous et al. 2021; Guo et al. 2024; Jiang et al. 2024), with an increased sampling in *T. betulae*, as the closest relative to our isolates. *Kwoniella mangrovensis* was used as an outgroup based on Jiang et al. (2024). The alignment included 32 terminals with 1096 characters—180 of which were parsimony-informative and 840, constant. The substitution model TIM2e + FQ + G4 was selected for the ITS1 and the ITS2, and the TNe + FQ + I + G4 for the 5.8S and the LSU. We considered four independent partitions, ITS1 (1-171), 5.8S (172-327), ITS2 (328-538) and LSU (539-1096). Maximum likelihood bootstrap values ≥ 70% are indicated below branches. The isolates obtained in this work are highlighted in bold.

Additional file13 **Fig. S11** Phylogenetic relationships of yeast isolates obtained from *Peltigera* and related taxa in the genus *Vishniacozyma (Tremellomycetes)*. The dataset included a fair representation of the species accepted in the genus (Liu et al. 2015b), with an increased sampling in *V. carnescens*, *V. dimennae*, *V. globispora*, *V. tephrensis* and *V. victoriae* as the closest relatives to our isolates. *Tremella flava* was used as an outgroup based on Liu et al. (2015b). The alignment included 49 terminals with 507 characters—101 of which were parsimony-informative and 355, constant. The substitution model TIM2e + FQ + I + G4 was selected for ITS1 and ITS2, and the JC for the 5.8S. We considered three independent partitions, ITS1 (1-75), 5.8S (76-185) and ITS2 (186-507). Maximum likelihood bootstrap values ≥ 70% are indicated below branches. The isolates obtained in this work are highlighted in bold.

## Data Availability

The sequences obtained in this study were deposited in the GenBank database with accession numbers OQ448325 to OQ448503 (ITS) and PQ362932 to PQ362992 (LSU). The occurrence data and metadata of the isolates are accessible through GBIF: https://doi.org/10.15468/hw7k9r. The type strains for each species described in this study are preserved in a metabolically inactive state in the Chilean Collection of Microbial Genetic Resources (CChRGM) https://www.cchrgm.cl/.

## Abbreviations

AICc: Corrected Akaike information criterion
CBS: Centraalbureau voor Schimmelcultures
CChRGM: Chilean Collection of Microbial Genetic Resources
°C: Celsius degree
DGD: Dehydrated gelatin drop
DNA: Deoxyribonucleic acid
EDTA: Ethylenediaminetetraacetic acid
e.g.: *Exempli gratia* (For example)
g: Grams
i.e: *id est* (That is)
ITS: Internal transcribed spacer
JCM: Japan collection of microorganisms
LSU: Nuclear large subunit ribosomal
ML: Maximum likelihood
µl: Microliter
µg: Microgram
ml: Milliliter
mM: Millimolar
min: Minute
NaCl: Sodium chloride
PDA: Potato dextrose agar
pH: Potential of hydrogen
pro term: *pro tempore* (Species names that are temporarily maintained)
rpm: Revolutions per minute
s: Seconds
s. l.: Sensu lato (Broad sense)
TE: Tris-EDTA buffer
USA: United States of America
viz: *videre licet* (Namely)
v/v: Volume/volume
w/v: Weight/volume
YMA: Yeast Malt Agar
YNB: Yeast nitrogen base

## Declarations

### Adherence to national and international regulations

All necesary permits were obtained for the lichen sampling. All basidiomycete yeast isolates are maintained in culture collections.
