# Supplementary material for: Peltigera lichens as sources of uncharacterized cultured basidiomycete yeasts
Source: IMA Fungus. 2024 Dec 4;15:39. doi: 10.1186/s43008-024-00170-9 (PMC11616168; doi:10.1186/s43008-024-00170-9)
Supplement: Supplementary file 2 — Additional file2 Tables S2-S5 Physiological characteristics of the new species described in this study [file 43008_2024_170_MOESM2_ESM.docx]

**Table S2:** Physiological characteristics of the species belonging to the genus *Boekhoutia* (according to Li et al., 2020^1^; Jiang et al., 2024^2^*;* this study). Acronyms stand for: +: Growth; -: No growth; D: Delayed growth; V: variable; W: Weak growth and NR: not reported. *Boekhoutia peltigerae* sp. nov., investigated in this study, is highlighted in bold font.

| **Species** | ***Boekhoutia peltigerae* sp. nov.** | | *B. sterigmata*^1^ | *B. foliicola^2^* |
| --- | --- | --- | --- | --- |
| **Collection code** | **RGM 3670 (C23 B28)** | C23 B30 | CGMCC 2.4539 | CGMCC 2.6878 |
| **Carbon assimilation** |  |  |  |  |
| sodium citrate | - | - | - | NR |
| D-arabinose | D | + | - | - |
| L-arabinose | - | - | - | **+** |
| D-gluconate | D | D | NR | NR |
| D-mannitol | W | W | - | **+** |
| D-xylose | **-** | NR | - | W |
| i-erythritol | **-** | **-** | - | **-** |
| ethanol | **-** | **-** | - | D/W |
| D-galactose | **+** | **+** | + | + |
| D-glucose | **+** | **+** | **+** | **+** |
| glycerol | **+** | **-** | - | **-** |
| meso-inositol | NR | - | NR | NR |
| lactose | **-** | **-** | - | **-** |
| L-rhamnose | **+** | D | - | - |
| maltose | **+** | W | D/W | **+** |
| melibiose | **-** | **-** | - | **-** |
| methanol | **-** | **-** | - | **-** |
| N-acetyl-D-glucosamine | **-** | - | - | **-** |
| D-raffinose | **+** | **-** | - | **+** |
| sucrose | **+** | **+** | + | **+** |
| cellobiose | D | **-** | + | **+** |
| **Nitrogen assimilation** |  |  |  |  |
| ammonium sulfate | **+** | D | + | **+** |
| creatine | NR | W | NR | NR |
| creatinine | + | + | NR | NR |
| potassium nitrate | + | + | - | + |
| sodium nitrite | - | - | - | W |
| **Fermentation** |  |  |  |  |
| glucose | - | - | - | - |
| **Additional Tests** |  |  |  |  |
| 10% NaCl 5% glucose medium | - | - | NR | NR |
| starch synthesis (derivatives) | - | - | - | - |
| **Temperature** |  |  |  |  |
| 4°C | + | + | NR | NR |
| 10°C | + | + | NR | NR |
| 15°C | + | + | NR | NR |
| 30°C | - | - | - | - |

Jiang Y-L, Bao W-J, Liu F, et al (2024) Proposal of one new family, seven new genera and seventy new basidiomycetous yeast species mostly isolated from Tibet and Yunnan provinces, China. Stud Mycol 153:57–153. https://doi.org/10.3114/sim.2024.109.02

Li A, Yuan F, Groenewald M, et al (2020) Diversity and phylogeny of basidiomycetous yeasts from plant leaves and soil: Proposal of two new orders, three new families, eight new genera and one hundred and seven new species. Stud Mycol 96:17–140. https://doi.org/10.1016/j.simyco.2020.01.002

**Table S3:** Physiological characteristics of the species belonging to the genus *Cystobasidium* (according to Jiang et al., 2024^1^; Yurkov et al., 2015^2^; Fotedar et al., 2019^3^; this study). Acronyms stand for: +: Growth; -: No growth; D: Delayed growth; V: variable; W: Weak growth and NR: not reported. *Cystobasidium chilense* sp. nov. investigated in this study, is highlighted in bold font. These results of carbon assimilation C07_B20^T^ were obtained from YT MicroPlate (Biolog) and nitrogen assimilation and glucose fermentation, according to Kurtzman et al., 2011.

| **Species** | ***Cystobasidium chilense*** **sp.** **nov.** | *C. cunninghamiae*^1^ | *C. psychroaquaticum*^2^ | *C. lysinophilum* ^2,3^ |
| --- | --- | --- | --- | --- |
| **Collection code** | **RGM 3661 (C07 B20)** | XSR28-13 (=CGMCC 2.6841) | K-833 (=DSM 27713) | JCM 5951 (=CBS 9126) |
| **Carbon assimilation** |  |  |  |  |
| sodium citrate | - | NR | - | NR |
| citric acid | - | - | - | NR |
| D-arabinose | W | W | V | + |
| L-arabinose | **+** | V | + | NR |
| D-mannitol | **+** | + | + | NR |
| D-xylose | + | D | + | NR |
| i-erythritol | V | - | - | + |
| D-galactose | V | - | - | NR |
| D-glucose | **+** | + | + | NR |
| glycerol | **+** | + | + | NR |
| myo-inositol | - | - | - | - |
| lactose | - | - | - | + |
| L-rhamnose | V | - | NR | NR |
| maltose | W | - | - | + |
| maltriose | **+** | NR | NR | NR |
| melibiose | W | - | - | NR |
| melezitose | **+** | + | + | + |
| sucrose | **+** | + | + | + |
| cellobiose | **+** | - | V | + |
| D-glucosamine | V | - | NR | - |
| N-acetyl-D-glucosamine | + | D | NR | NR |
| D- ribose | W | V | V | NR |
| D-sorbitol (D-glucitol) | + | - | NR | NR |
| adonitol | + | NR | NR | NR |
| D-arabitol | + | NR | NR | NR |
| inulin | **+** | + | NR | NR |
| D-raffinose | W | - | NR | + |
| salicin | **+** | + | + | - |
| L-sorbose | **+** | + | V | - |
| fumaric acid | **+** | NR | NR | NR |
| L-malic acid | **+** | NR | NR | NR |
| succinic acid monomethyl ester | V | NR | NR | NR |
| bromosuccinic acid | **+** | NR | NR | NR |
| L-glutamic acid | **+** | NR | NR | NR |
| γ-aminobutyric acid | + | NR | NR | NR |
| α-ketoglutaric acid | + | NR | NR | NR |
| 2-keto-gluconic acid | + | NR | + | NR |
| D-gluconic acid | + | NR | NR | NR |
| dextrin | + | NR | NR | NR |
| gentiobiose | + | NR | NR | NR |
| palatinose | W | NR | NR | NR |
| stachyose | W | NR | NR | NR |
| D-trehalose | + | + | + | NR |
| turanose | + | NR | NR | NR |
| D-psicose | + | NR | NR | NR |
| α-methyl-D-glucoside | V | - | - | NR |
| β-methyl-D-glucoside | + | NR | NR | NR |
| amygdalin | + | NR | NR | NR |
| arbutin | + | NR | + | NR |
| salicin | + | NR | NR | NR |
| maltitol | **+** | NR | NR | NR |
| xylitol | **+** | NR | NR | NR |
| methyl succinate + D-xylose | + | NR | NR | NR |
| N-acetyl-L-glutamic acid + D-xylose | + | NR | NR | NR |
| quinolic acid + D-xylose | + | NR | NR | NR |
| D-glucuronic acid + D-xylose | + | NR | NR | NR |
| dextrin + D-xylose | + | NR | NR | NR |
| D-melibiose + D-xylose | + | NR | NR | NR |
| D-galactose + D-xylose | + | NR | NR | NR |
| m-inositol + D-xylose | + | NR | NR | NR |
| 1,2-propanediol + D-xylose | + | NR | NR | NR |
| acetoin + D-xylose | + | NR | NR | NR |
| **Nitrogen assimilation** |  |  |  |  |
| ammonium sulfate | + | + | NR | NR |
| creatine | W | NR | - | NR |
| potassium nitrate | + | + | - | - |
| sodium nitrite | - | + | - | NR |
| **Fermentation** |  |  |  |  |
| glucose | - | - | - | NR |
| **Additional Test** |  |  |  |  |
| synthesis of extracellular amyloids | - | - | NR | NR |
| **Temperature** |  |  |  |  |
| 4°C | + | NR | NR | NR |
| 10°C | + | NR | NR | NR |
| 15°C | + | NR | NR | NR |
| 25°C | + | + | + | + |
| 30°C | - | - | - | + |
| 37°C | - | - | - | - |

Jiang Y-L, Bao W-J, Liu F, et al (2024) Proposal of one new family, seven new genera and seventy new basidiomycetous yeast species mostly isolated from Tibet and Yunnan provinces, China. Stud Mycol 153:57–153. https://doi.org/10.3114/sim.2024.109.02

Yurkov, A. M., Kachalkin, A. V., Daniel, H. M., Groenewald, M., Libkind, D., de Garcia, V., ... & Begerow, D. (2015). Two yeast species *Cystobasidium psychroaquaticum* fa sp. nov. and *Cystobasidium rietchieii* fa sp. nov. isolated from natural environments, and the transfer of *Rhodotorula minuta* clade members to the genus *Cystobasidium*. Antonie Van Leeuwenhoek, 107, 173-185. https://doi.org/10.1007/s10482-014-0315-0

Fotedar R, Fell JW, Boekhout T, et al (2019) *Cystobasidium halotolerans* sp. nov., a novel basidiomycetous yeast species isolated from the Arabian Gulf. Int J Syst Evol Microbiol 69:839–845. https://doi.org/10.1099/ijsem.0.003250

**Table S4:** Physiological characteristics of the species belonging to the genus *Genolevuria* (according to Inácio et al., 2005^1^; Landell et al., 2009^2^; this study). Acronyms stand for: +: Growth; -: No growth; D: Delayed growth; V: variable; W: Weak growth and NR: not reported. In bold is the type isolate of each genus. The results were obtained through classical tests, according to Kurtzman et al., 2011.

| **Species** | ***Genolevuria patagoniensis* sp. nov.** | *G. armeniaca*^1^ | *G. bromeliarum^2^* |
| --- | --- | --- | --- |
| **Sinonym** | ***-*** | *Cryptococcus armeniacus* | *C. bromeliarum* |
| **Collection code** | **RGM 3631 (T20 B01)** | CBS 10050 | CBS 10424 = BI20 |
| **Carbon assimilation** |  |  |  |
| sodium citrate | - | D | - |
| D-arabinose | + | D | + |
| L-arabinose | + | + | + |
| D-gluconate | D | NR | NR |
| D-mannitol | V | D | + |
| D-xylose | + | + | + |
| i-erythritol | - | - | NR |
| ethanol | V | - | - |
| D-galactose | + | D | + |
| D-glucose | + | + | + |
| glycerol | D | W | V |
| meso-inositol | + | NR | NR |
| lactose | + | + | + |
| L-rhamnose | + | D | + |
| maltose | + | + | + |
| melibiose | + | + | + |
| methanol | - | - | - |
| N-acetyl-D-glucosamine | D | NR | + |
| D-raffinose | D | + | + |
| sucrose | + | + | + |
| cellobiose | + | + | V |
| **Nitrogen assimilation** |  |  |  |
| ammonium sulfate | D | NR | NR |
| creatine | + | - | - |
| creatinine | + | - | - |
| potassium nitrate | + | - | NR |
| sodium nitrite | - | - | V |
| **Fermentation** |  |  |  |
| glucose | - | - | - |
| **Additional Test** |  |  |  |
| Vitamins free medium | - | - | NR |
| 10% NaCl 5% Glucose | - | NR | - |
| Extracellular amyloid synthesis | - | W | - |
| Agar 50 % glucose-yeast extract yeast extract | - | NR | - |
| **Temperature** |  |  |  |
| 4°C | + | NR | NR |
| 10°C | + | NR | NR |
| 15°C | + | NR | NR |
| 25°C | + | + | NR |
| 30°C | - | - | NR |

Inácio, J., Portugal, L., Spencer-Martins, I., & Fonseca, Á. (2005). Phylloplane yeasts from Portugal: Seven novel anamorphic species in the *Tremellales* lineage of the *Hymenomycetes* (*Basidiomycota*) producing orange-coloured colonies. *FEMS Yeast Research*, *5*(12), 1167–1183. https://doi.org/10.1016/j.femsyr.2005.05.007

Landell, M. F., Inacio, J., Fonseca, A., Vainstein, M. H., & Valente, P. (2009). *Cryptococcus bromeliarum* sp. nov., an orange-coloured basidiomycetous yeast isolated from bromeliads in Brazil. International journal of systematic and evolutionary microbiology, 59(4), 910-913. https://doi.org/10.1099/ijs.0.005652-0

**Table S5:** Physiological characteristics of the species belonging to the genus *Pseudotremella* (according to Bandoni and Boekhout, 2001^1^; Middelhoven, 2005^2^; Satoh et al., 2013^3^). Acronyms stand for: +: Growth; -: No growth; D: Delayed growth; V: variable; W: Weak growth and NR: not reported and Th, thiamine required. CBS: Fungal Biodiversity Center. *Pseudotremella navarinensis* sp. nov., investigated in this study, is highlighted in bold font.

| **Species** | ***Pseudotremella navarinensis*** **sp. nov.** | *P. moriformis*^1^ | *P. allantoinivorans^2^* | *P. lacticolor*^3^ |
| --- | --- | --- | --- | --- |
| **Synonym** | **-** | *Tremella moriformis* | *Cryptococcus allantoinivorans* | *Cryptococcus lacticolor* |
| **Collection code** | **RGM 3659 (N129 B01)** | CBS 7810 | CBS 9604 | CBS 10915 |
| **Carbon assimilation** |  |  |  |  |
| sodium citrate | D | + | + | W |
| D-arabinose | + | D | + | + |
| L-arabinose | + | + | + | + |
| D-gluconate | + | + | + | + |
| D-mannitol | + | + | + | + |
| D-xylose | + | + | + | + |
| i-erythritol | + | - | NR | + |
| ethanol | + | - | + | W |
| D-galactose | + | + | + | + |
| D-glucose | + | + | + | + |
| glycerol | + | D | + | W |
| meso-inositol | + | NR | NR | NR |
| lactose | + | W | D | W |
| L-rhamnose | + | D | + | + |
| maltose | + | + | + | + |
| melibiose | + | - | + | W |
| methanol | - | - | - | - |
| N-acetyl-D-glucosamine | + | NR | + | + |
| D-raffinose | D | D | + | + |
| sucrose | + | + | + | + |
| cellobiose | + | D | + | + |
| **Nitrogen assimilation** |  |  |  |  |
| ammonium sulfate | D | NR | NR | + |
| creatinine | + | NR | + | NR |
| creatine | + | NR | D | NR |
| potassium nitrate | + | NR | - | - |
| sodium nitrite | - | W | - | - |
| **Fermentation** |  |  |  |  |
| glucose | - | - | - | - |
| **Additional Test** |  |  |  |  |
| Vitamin-free medium | + | - | Th | + |
| 10% NaCl 5% glucose medium | W | NR | + | NR |
| 50 % glucose-yeast extract agar | + | - | NR | - |
| synthesis of extracellular amyloids | - | + | + | + |
| **Temperature** |  |  |  |  |
| 4°C | + | NR | NR | NR |
| 10°C | + | NR | NR | NR |
| 15°C | + | NR | NR | NR |
| 25°C | + | + | NR | NR |
| 30°C | - | D | + | + |

Bandoni, R. G., Boekhout, T. 2011. Tremella Peerson (1794). In: Kurtzman, C.P., Fell, J.W., Boekhout, T. (Eds.), The Yeasts: A Taxonomic Study. Elsevier, London, pp. 1567–1590.

Middelhoven, W. J. (2005). *Cryptococcus allantoinivorans* sp.nov., an anamorphic basidiomycetous yeast (*Tremellales*) physiologically resembling other species of the *Cryptococcus laurentii* complex that degrade polysaccharides and C2 compounds. *Antonie van Leeuwenhoek*, *87*(2), 101–108. https://doi.org/10.1007/s10482-004-1728-y

Satoh, K., Maeda, M., Umeda, Y., Sugamata, M., & Makimura, K. (2013). *Cryptococcus lacticolor* sp. nov. and Rhodotorula oligophaga sp. nov., novel yeasts isolated from the nasal smear microbiota of Queensland koalas kept in Japanese zoological parks. *Antonie van Leeuwenhoek, International Journal of General and Molecular Microbiology*, *104*(1), 83–93. https://doi.org/10.1007/s10482-013-9928-y
