# Supplementary figures and images for: Peltigera lichens as sources of uncharacterized cultured basidiomycete yeasts
Source: IMA Fungus. 2024 Dec 4;15:39. doi: 10.1186/s43008-024-00170-9 (PMC11616168; doi:10.1186/s43008-024-00170-9)

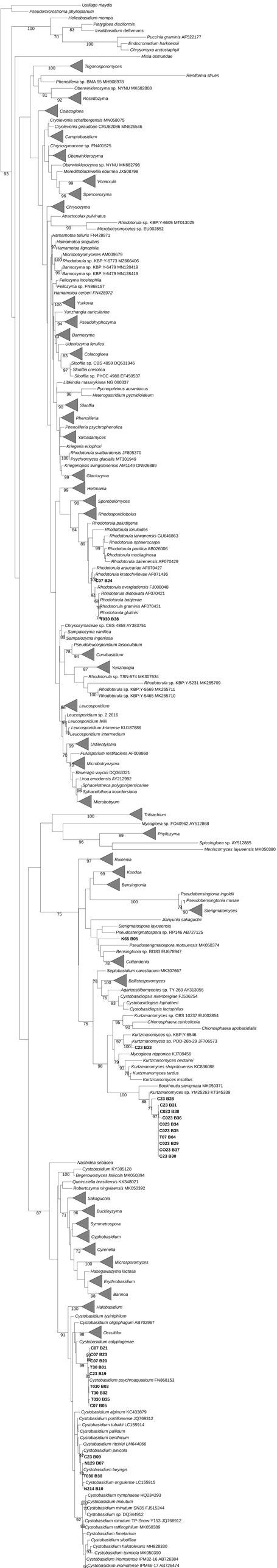

Supplement: Supplementary file 3 — Additional file3 Fig. S1 Phylogenetic relationships of yeast isolates obtained from Peltigera and related taxa in the subphylum Pucciniomycotina. The dataset was based on recently published phylogenetic trees (Jiang et al. 2024; Kachalkin et al. 2024) and included LSU sequences from our isolates from Peltigera and sequences representing the 11 accepted orders and four accepted classes in the group (Wang et al. 2015a, b). Two members of the Ustilaginomycotina, viz. Pseudomicrostroma phylloplanumand Mycosarcoma (=Ustilago) maydis were used as outgroups based on Wang et al. (2015b). The alignment included 506 sequences with 680 characters—431 of which were parsimony-informative and 174, constant. We considered only one partition corresponding to the LSU. The substitution model GTR + F + I + Γ4 was selected. Maximum likelihood bootstrap values ≥70% are indicated below branches. The isolates obtained in this work are highlighted in bold. [file 43008_2024_170_MOESM3_ESM.pdf]

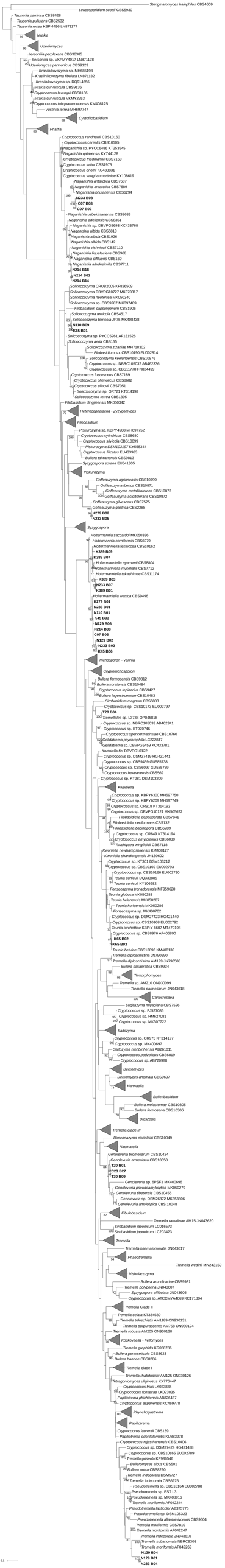

Supplement: Supplementary file 4 — Additional file4 Fig. S2 Phylogenetic relationships of yeast isolates obtained from Peltigera and related taxa in the class Tremellomycetes. The dataset included LSU sequences from our isolates from Peltigera, and sequences of most genera representing the 17 families of the five accepted orders in the class (Liu et al. 2015a, b). Sterigmatomyces halophilus (Agaricostilbomycetes, Pucciniomycotina) was used as an outgroup based on Liu et al. (2015b). The alignment included 667 sequences with 654 characters—354 of which were parsimony-informative and 205, constant. We considered only one partition corresponding to the LSU. The substitution model SYM + I + Γ4 was selected. Maximum likelihood bootstrap values ≥ 70% are indicated below branches. The isolates obtained in this work are highlighted in bold. [file 43008_2024_170_MOESM4_ESM.pdf]

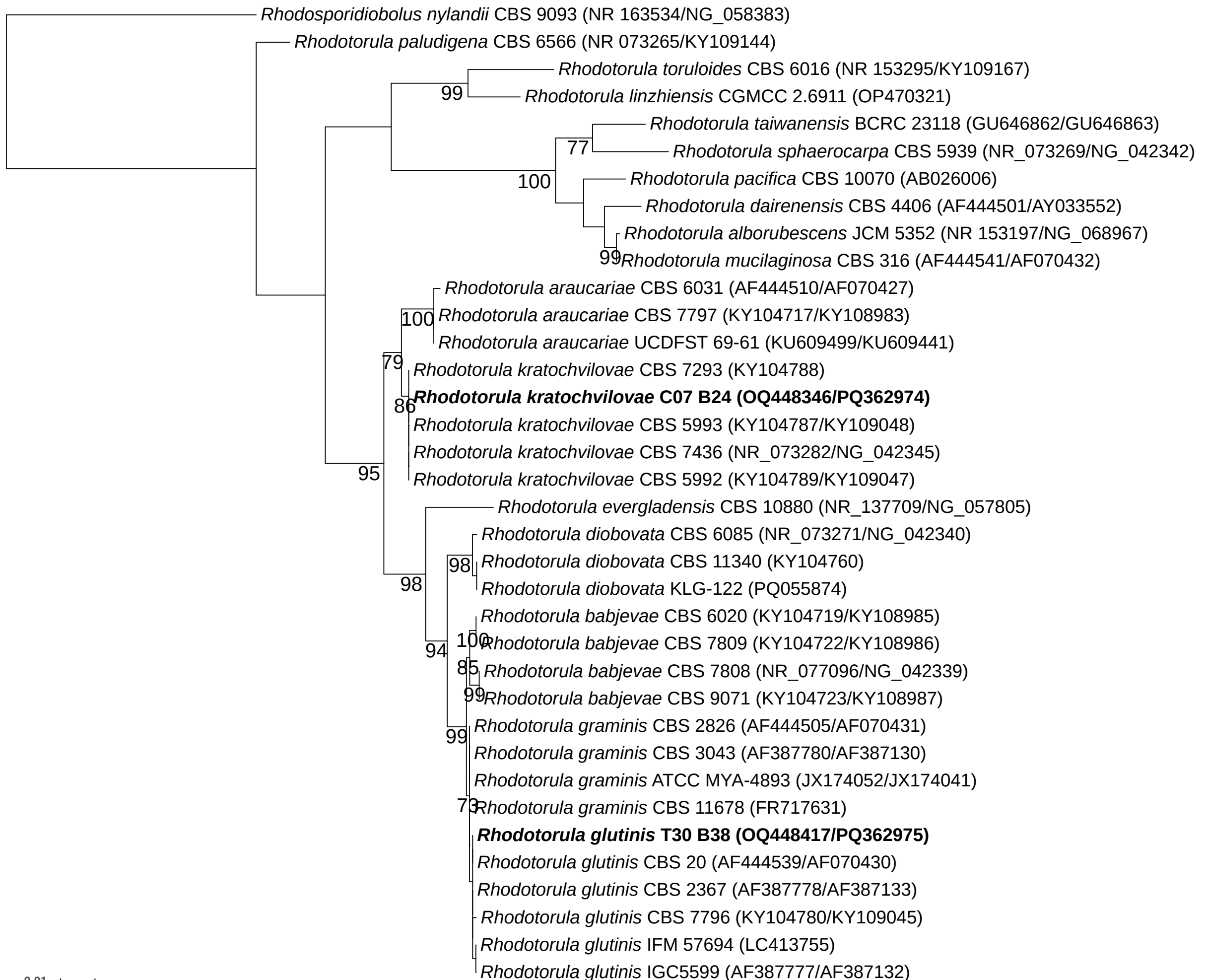

Supplement: Supplementary file 5 — Additional file5 Fig. S3 Phylogenetic relationships of yeast isolates obtained from Peltigera and related taxa in the genus Rhodotorula (Pucciniomycotina). The dataset included a fair representation of the species accepted in the genus (Wang et al. 2015b; Jiang et al. 2024), with an increased sampling in R. araucariae, R. babjevae, R. diobovata, R. evergladensis, R. glutinis, R. graminis, and R. kratochvilovae, as the closest relatives to our isolates. Rhodosporidiobolus nylandii was used as an outgroup based on Wang et al. (2015a). The alignment included 37 terminals with 1208 characters—150 of which were parsimony-informative and 972, constant. The substitution model K3Pu + F + G4 was selected for the ITS1 and the ITS2, and the TPM3 + FQ + I for the 5.8S and for the LSU. We considered four independent partitions: ITS1 (1-198), 5.8S (199-358), ITS2 (359-574), and LSU (575-1208). Maximum likelihood bootstrap values ≥ 70% are indicated below branches. The isolates obtained in this work are highlighted in bold. [file 43008_2024_170_MOESM5_ESM.pdf]

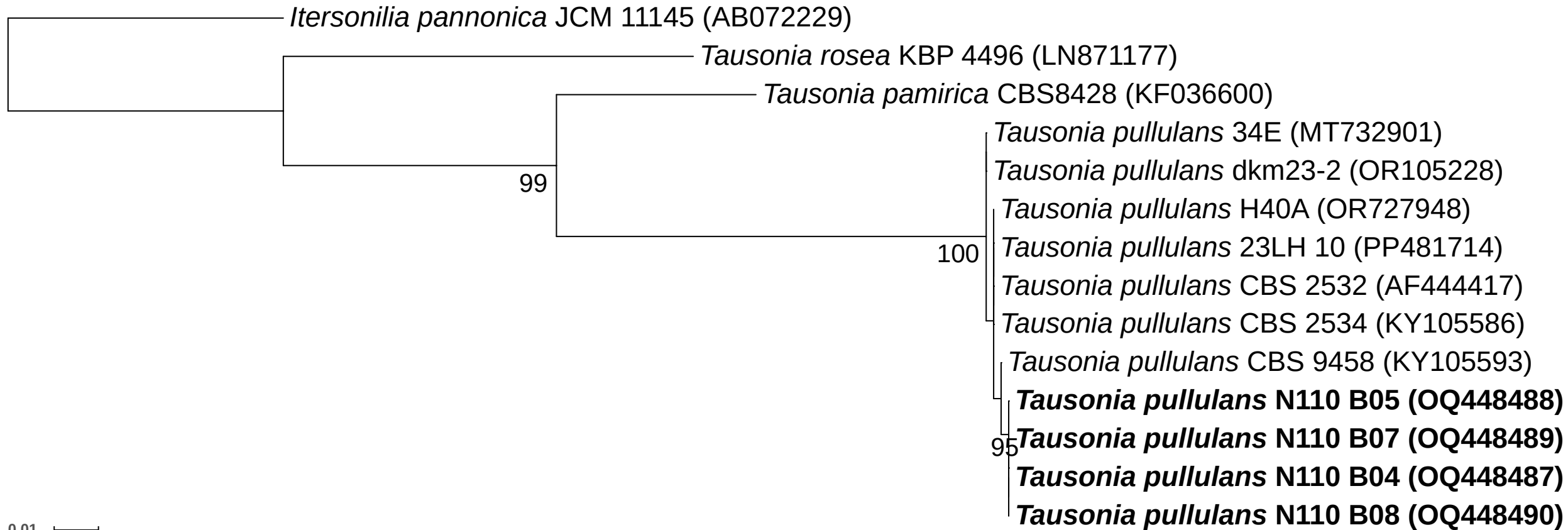

Supplement: Supplementary file 6 — Additional file6 Fig. S4 Phylogenetic relationships of yeast isolates obtained from Peltigera and related taxa in the genus Tausonia (Tremellomycetes). The dataset included sequences of all species accepted in the genus, with an increased sampling in T. pullulans as the closest relative to our isolates. Itersonilia pannonica was used as an outgroup based on Kachalkin et al. (2019). The alignment included 14 terminals with 639 characters—54 of which were parsimony-informative and 498, constant. The substitution model TIMe + FQ + G4 was selected for ITS1 and ITS2, and the JC for the 5.8S. We considered three independent partitions, ITS1 (1-179), 5.8S (180-354), and ITS2 (355-639). Maximum likelihood bootstrap values ≥ 70% are indicated below branches. The isolates obtained in this work are highlighted in bold. [file 43008_2024_170_MOESM6_ESM.pdf]

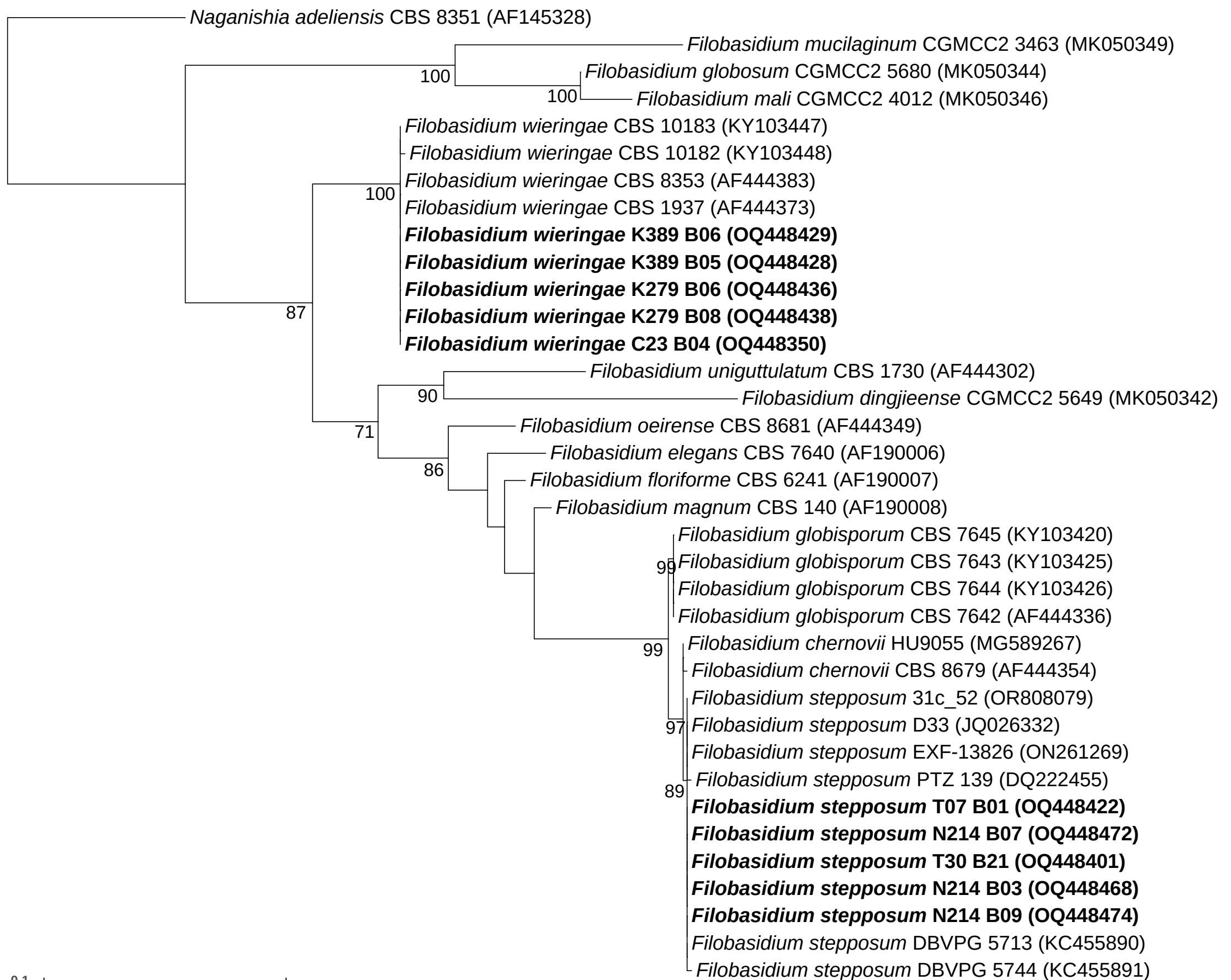

Supplement: Supplementary file 7 — Additional file7 Fig. S5 Phylogenetic relationships of yeast isolates obtained from Peltigera and related taxa in the genus Filobasidium (Tremellomycetes). The dataset included all sequenced species in the genus (Li et al. 2020), except for F. chaidanense, which was otherwise not closely related to our isolates (Wei et al. 2022). Naganishia adeliensis was used as an outgroup based on Li et al. (2020). The alignment included 36 terminals with 607 characters—142 of which were parsimony-informative and 399, constant. The substitution model HKY + F + G4 was selected for ITS1 and ITS2, and the JC for the 5.8S. We considered three independent partitions, ITS1 (1-182), 5.8S (183-330), and ITS2 (331-607). Maximum likelihood bootstrap values ≥ 70% are indicated below branches. The isolates obtained in this work are highlighted in bold. [file 43008_2024_170_MOESM7_ESM.pdf]

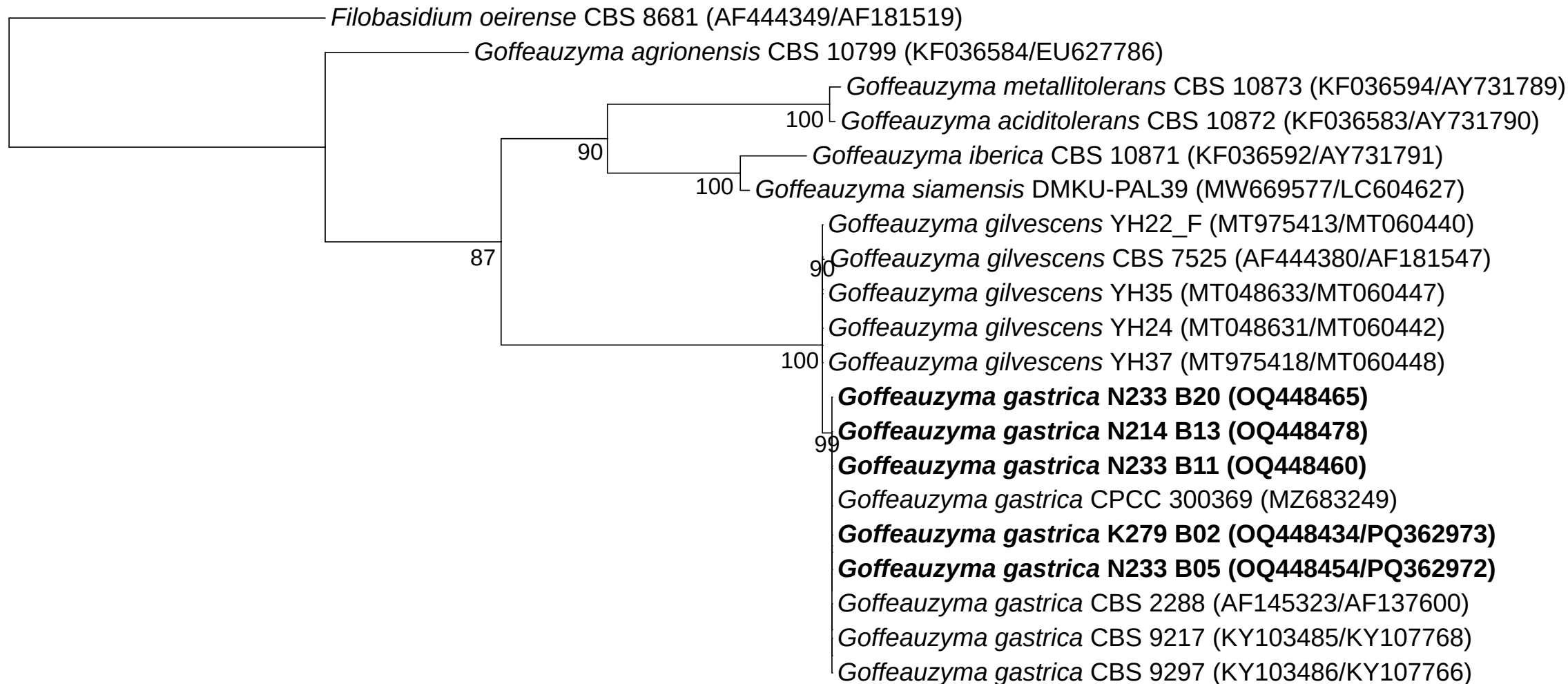

0.1

Supplement: Supplementary file 8 — Additional file8 Fig. S6 Phylogenetic relationships of yeast isolates obtained from Peltigera and related taxa in the genus Goffeauzyma (Tremellomycetes). The dataset included sequences of all species accepted in the genus (Liu et al. 2015b; Nutaratat et al. 2022), with an increased sampling in G. gastrica and G. gilvescens, as the closest relatives to our isolates. Filobasidium oeirense was used as an outgroup based on Liu et al. (2015b). The alignment included 20 terminals with 1230 characters—211 of which were parsimony-informative and 915, constant. The substitution model TIM2 + F + G4 was selected for the ITS1 and the ITS2, and the K2P + FQ + I for the 5.8S and the LSU. We considered four independent partitions: ITS1 (1-224), 5.8S (225-385), ITS2 (386-653), and LSU (654-1230). Maximum likelihood bootstrap values ≥ 70% are indicated below branches. The isolates obtained in this work are highlighted in bold. [file 43008_2024_170_MOESM8_ESM.pdf]

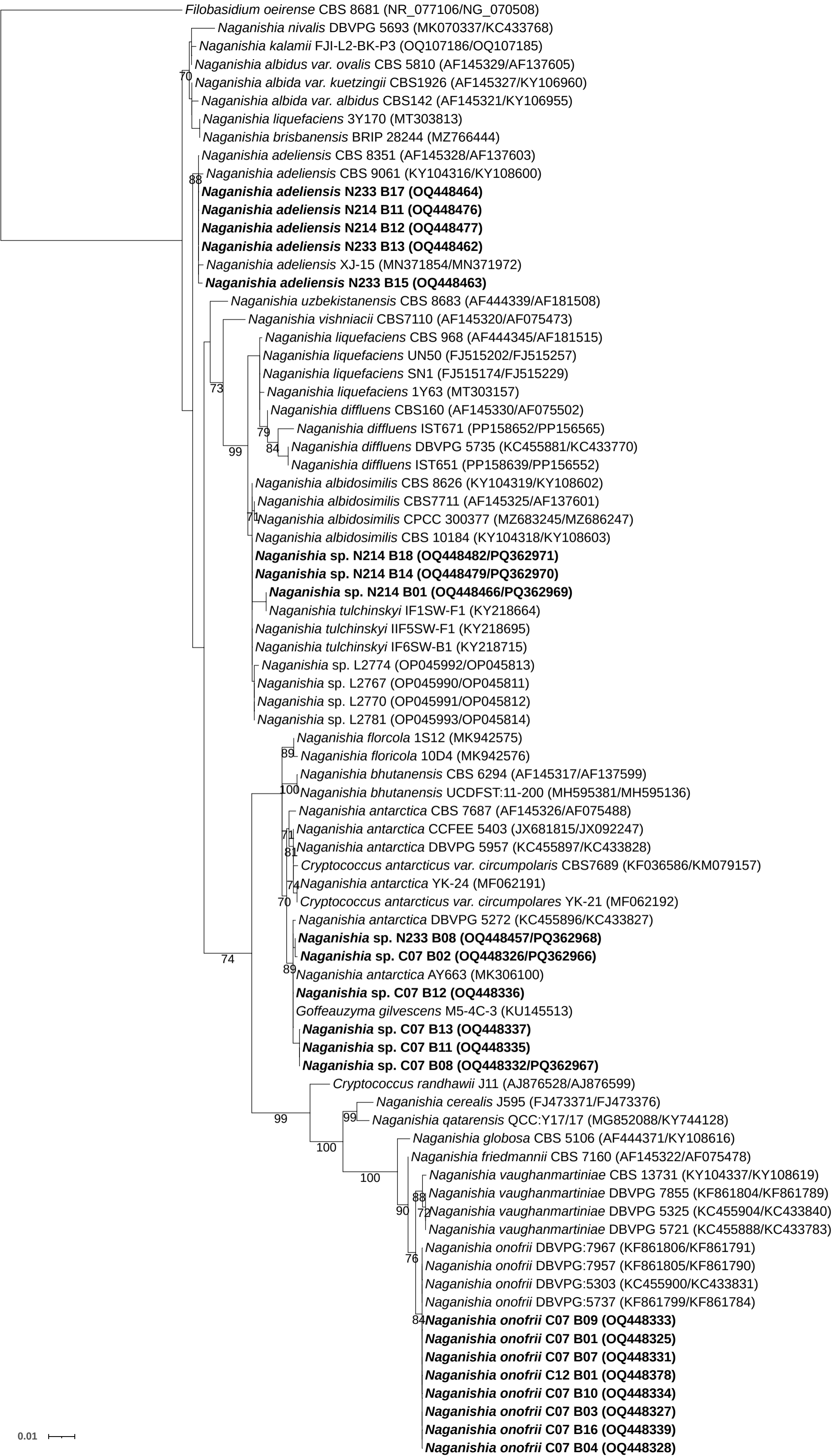

Supplement: Supplementary file 9 — Additional file9 Fig. S7 Phylogenetic relationships of yeast isolates obtained from Peltigera and related taxa in the genus Naganishia (Tremellomycetes). The dataset included all sequenced species in the genus (Liu et al. 2015b; Bijlani et al. 2022; Leo et al. 2023), except for Naganishia indica, which was otherwise not related to our isolates (Crous et al. 2019). Filobasidium oeirense was used as an outgroup based on Liu et al. (2015b). The alignment included 80 terminals with 1354 characters—141 of which were parsimony-informative and 1099, constant. The substitution model K3P + FQ + I was selected for the ITS1, the F81 + F for the 5.8S, and the TIM2 + F + I + G4 for the ITS2 and the LSU. We considered four independent partitions: ITS1 (1-169), 5.8S (170-321), ITS2 (322-626), and LSU (627-1354). Maximum likelihood bootstrap values ≥ 70% are indicated below branches. The isolates obtained in this work are highlighted in bold. [file 43008_2024_170_MOESM9_ESM.pdf]

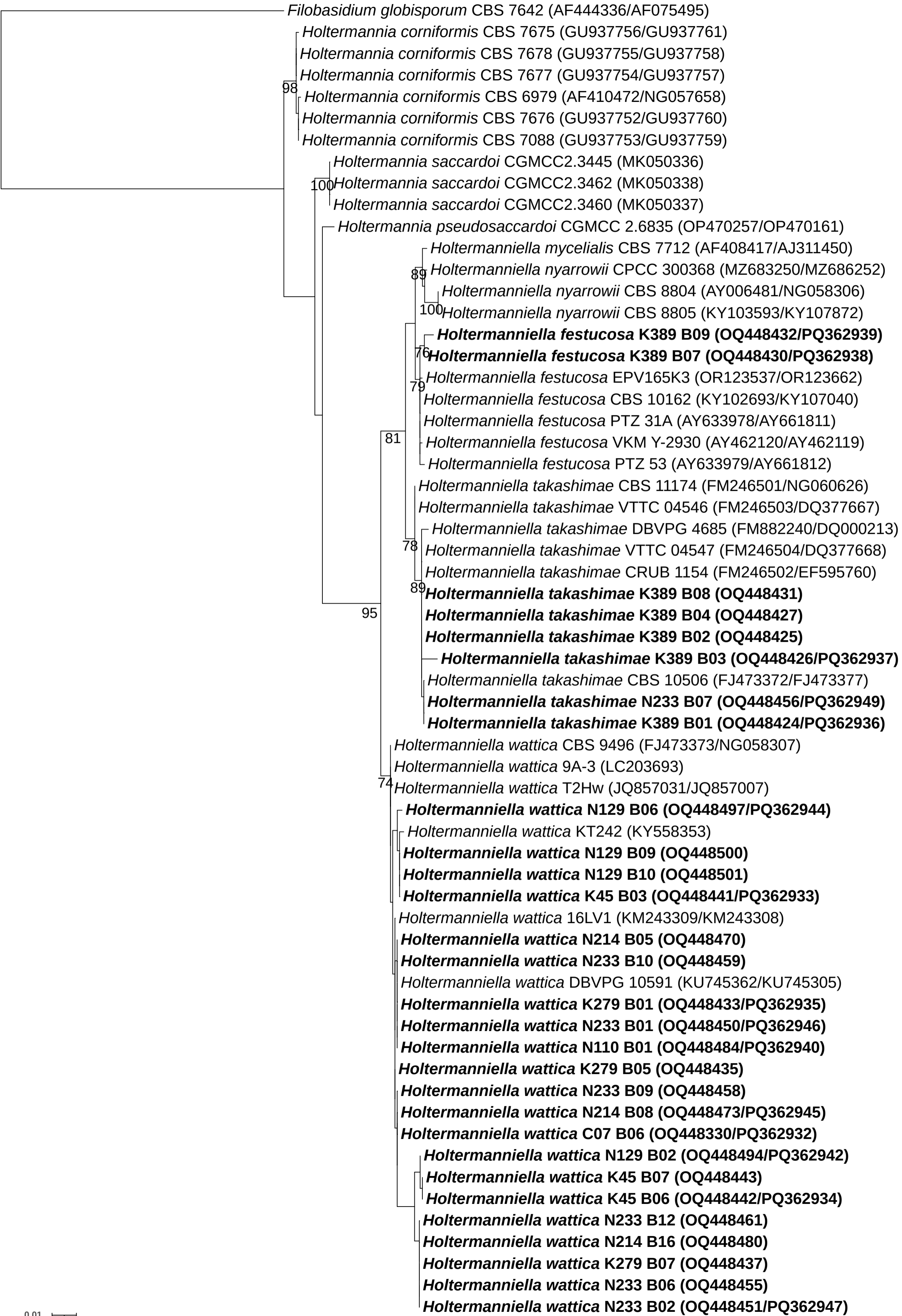

Supplement: Supplementary file 11 — Additional file11 Fig. S9 Phylogenetic relationships of yeast isolates obtained from Peltigera and related taxa in the genus Holtermanniales (Tremellomycetes). The dataset included sequences of Holtermannia and Holtermanniella, the two accepted genera in the order (Wuczkowski et al. 2011; Liu et al. 2015b; Jiang et al. 2024), with an extended sampling in Holtermaniella festucosa, H. takashimae and H. wattica, as the closest relatives to our isolates. Filobasidium globisporum was used as an outgroup based on Liu et al. (2015b). The alignment included 61 terminals with 1153 characters—83 of which were parsimony-informative and 934, constant. The substitution model TIM2e + FQ + I + G4 was selected for all partitions. We considered four independent partitions: ITS1 (1-143), 5.8S (144-313), ITS2 (314-545), and LSU (546-1153). Maximum likelihood bootstrap values ≥ 70% are indicated below branches. The isolates obtained in this work are highlighted in bold. [file 43008_2024_170_MOESM11_ESM.pdf]

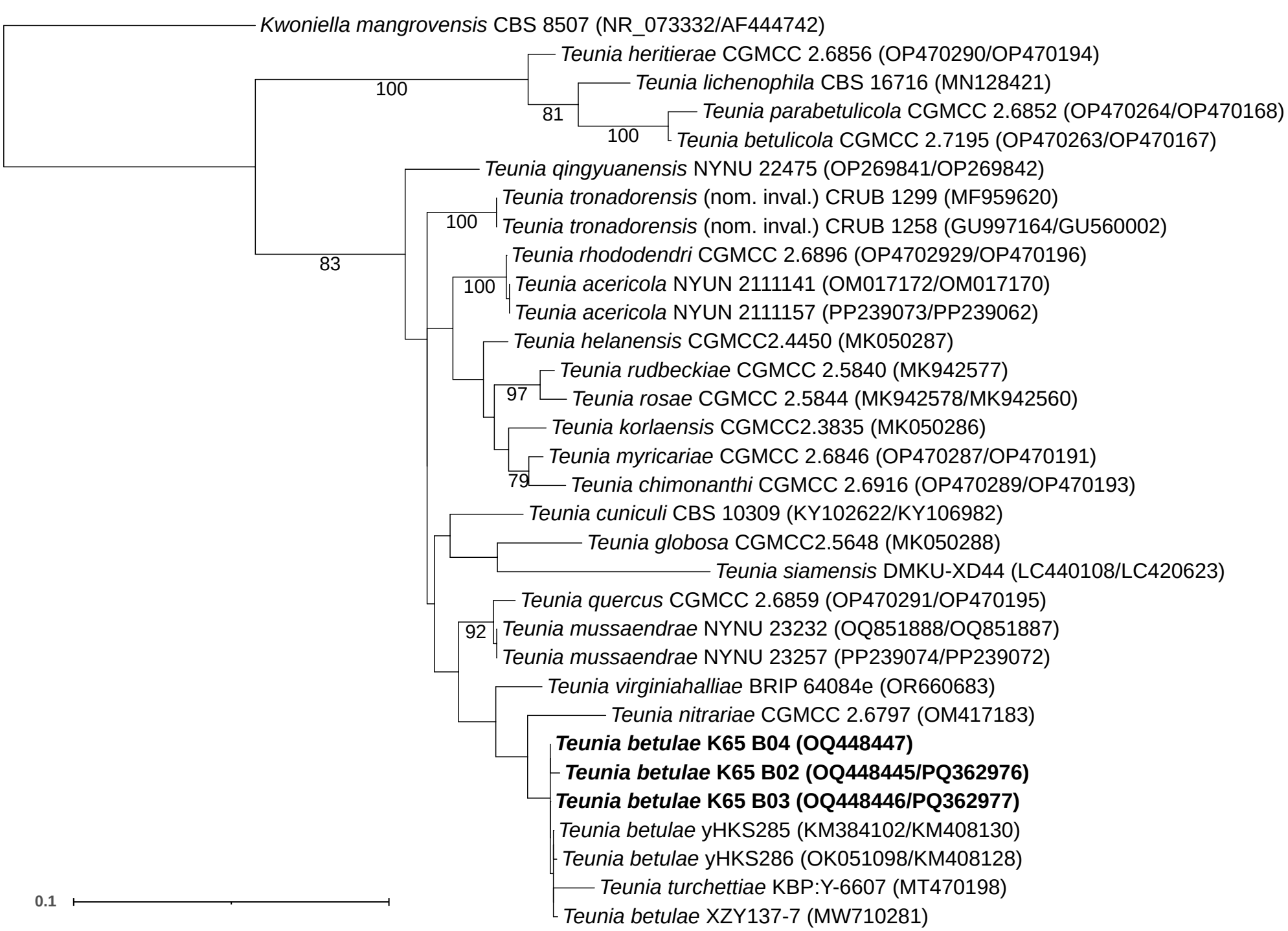

Supplement: Supplementary file 12 — Additional file12 Fig. S10 Phylogenetic relationships of yeast isolates obtained from Peltigera and related taxa in the genus Teunia (Tremellomycetes). The dataset included sequences of all species accepted in the genus (Li et al. 2020; Khunnamwong et al. 2020; Crous et al. 2021; Guo et al. 2024; Jiang et al. 2024), with an increased sampling in T. betulae, as the closest relative to our isolates. Kwoniella mangrovensis was used as an outgroup based on Jiang et al. (2024). The alignment included 32 terminals with 1096 characters—180 of which were parsimony-informative and 840, constant. The substitution model TIM2e + FQ + G4 was selected for the ITS1 and the ITS2, and the TNe + FQ + I + G4 for the 5.8S and the LSU. We considered four independent partitions, ITS1 (1-171), 5.8S (172-327), ITS2 (328-538) and LSU (539-1096). Maximum likelihood bootstrap values ≥ 70% are indicated below branches. The isolates obtained in this work are highlighted in bold. [file 43008_2024_170_MOESM12_ESM.pdf]

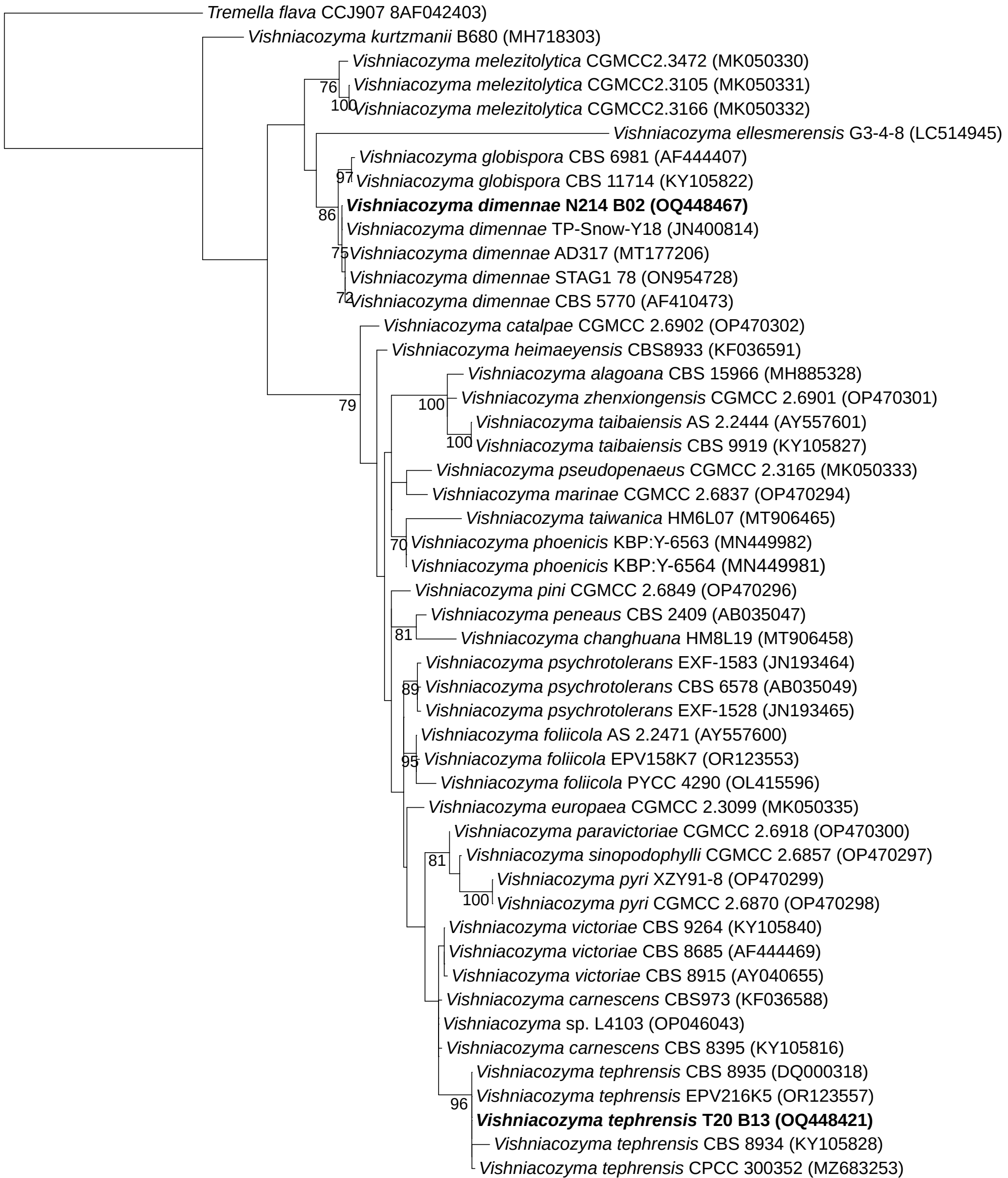

Supplement: Supplementary file 13 — Additional file13 Fig. S11 Phylogenetic relationships of yeast isolates obtained from Peltigera and related taxa in the genus Vishniacozyma (Tremellomycetes). The dataset included a fair representation of the species accepted in the genus (Liu et al. 2015b), with an increased sampling in V. carnescens, V. dimennae, V. globispora, V. tephrensis and V. victoriae as the closest relatives to our isolates. Tremella flava was used as an outgroup based on Liu et al. (2015b). The alignment included 49 terminals with 507 characters—101 of which were parsimony-informative and 355, constant. The substitution model TIM2e + FQ + I + G4 was selected for ITS1 and ITS2, and the JC for the 5.8S. We considered three independent partitions, ITS1 (1-75), 5.8S (76-185) and ITS2 (186-507). Maximum likelihood bootstrap values ≥ 70% are indicated below branches. The isolates obtained in this work are highlighted in bold. [file 43008_2024_170_MOESM13_ESM.pdf]
