# Supplementary material for: Peltigera lichens as sources of uncharacterized cultured basidiomycete yeasts
Source: IMA Fungus. 2024 Dec 4;15:39. doi: 10.1186/s43008-024-00170-9 (PMC11616168; doi:10.1186/s43008-024-00170-9)
Supplement: Supplementary file 10 — Additional file10 Fig. S8 Phylogenetic relationships of yeast isolates obtained from Peltigera and related taxa in the genus Solicoccozyma (Tremellomycetes). The dataset included sequences of all species accepted in the genus (Liu et al. 2015b, Yurkov and Kurtzman 2019; Li et al. 2020), with an increased sampling in S. aeria, S. fuscescens, S. gelidoterrea, S. phenolica, S. terrea, S. terricola, as the closest relatives to our isolates. Piskurozyma capsuligena was used as an outgroup based on Li et al. (2020). The alignment included 58 terminals with 661 characters—135 of which were parsimony-informative and 464, constant. The substitution model HKY + F + G4 was selected for ITS1 and ITS2, and the JC for the 5.8S. We considered three independent partitions, ITS1 (1-215), 5.8S (216-382), and ITS2 (383-661). Maximum likelihood bootstrap values ≥ 70% are indicated below branches. The isolates obtained in this work are highlighted in bold. [file 43008_2024_170_MOESM10_ESM.pdf]

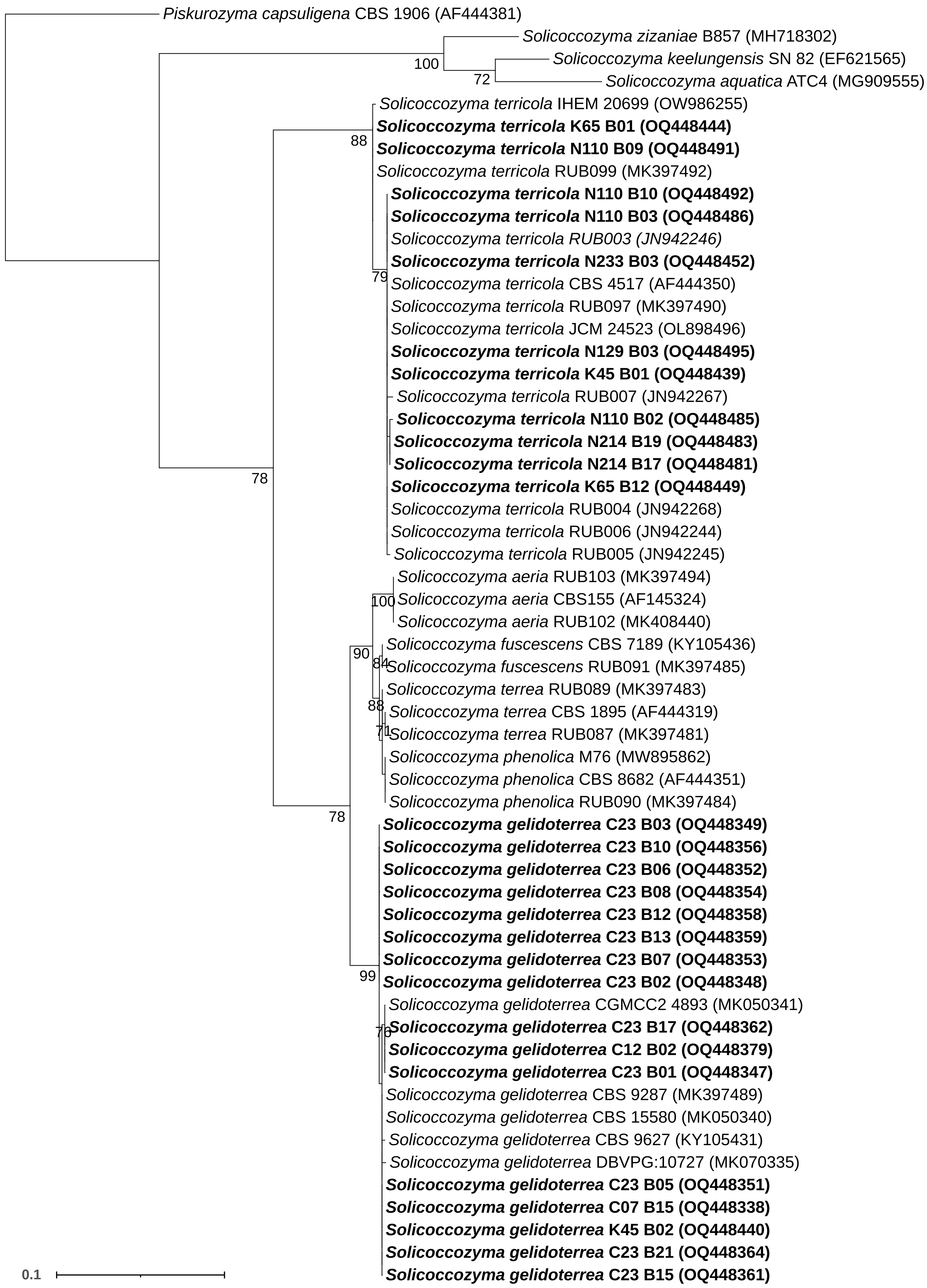

Piskurozyma capsuligena CBS 1906 (AF444381)
Solicoccozyma zizaniae B857 (MH718302)
100
Solicoccozyma keelungensis SN 82 (EF621565)
72
Solicoccozyma aquatica ATC4 (MG909555)
Solicoccozyma terricola IHEM 20699 (OW986255)
Solicoccozyma terricola K65 B01 (OQ448444)
88
Solicoccozyma terricola N110 B09 (OQ448491)
Solicoccozyma terricola RUB099 (MK397492)
Solicoccozyma terricola N110 B10 (OQ448492)
Solicoccozyma terricola N110 B03 (OQ448486)
Solicoccozyma terricola RUB003 (JN942246)
Solicoccozyma terricola N233 B03 (OQ448452)
79
Solicoccozyma terricola CBS 4517 (AF444350)
Solicoccozyma terricola RUB097 (MK397490)
Solicoccozyma terricola JCM 24523 (OL898496)
Solicoccozyma terricola N129 B03 (OQ448495)
Solicoccozyma terricola K45 B01 (OQ448439)
Solicoccozyma terricola RUB007 (JN942267)
Solicoccozyma terricola N110 B02 (OQ448485)
Solicoccozyma terricola N214 B19 (OQ448483)
Solicoccozyma terricola N214 B17 (OQ448481)
78
Solicoccozyma terricola K65 B12 (OQ448449)
Solicoccozyma terricola RUB004 (JN942268)
Solicoccozyma terricola RUB006 (JN942244)
Solicoccozyma terricola RUB005 (JN942245)
Solicoccozyma aeria RUB103 (MK397494)
100
Solicoccozyma aeria CBS155 (AF145324)
Solicoccozyma aeria RUB102 (MK408440)
Solicoccozyma fuscescens CBS 7189 (KY105436)
90
84
Solicoccozyma fuscescens RUB091 (MK397485)
Solicoccozyma terrea RUB089 (MK397483)
88
Solicoccozyma terrea CBS 1895 (AF444319)
71
Solicoccozyma terrea RUB087 (MK397481)
Solicoccozyma phenolica M76 (MW895862)
Solicoccozyma phenolica CBS 8682 (AF444351)
Solicoccozyma phenolica RUB090 (MK397484)
78
Solicoccozyma gelidoterrea C23 B03 (OQ448349)
Solicoccozyma gelidoterrea C23 B10 (OQ448356)
Solicoccozyma gelidoterrea C23 B06 (OQ448352)
Solicoccozyma gelidoterrea C23 B08 (OQ448354)
Solicoccozyma gelidoterrea C23 B12 (OQ448358)
Solicoccozyma gelidoterrea C23 B13 (OQ448359)
Solicoccozyma gelidoterrea C23 B07 (OQ448353)
99
Solicoccozyma gelidoterrea C23 B02 (OQ448348)
Solicoccozyma gelidoterrea CGMCC2 4893 (MK050341)
76
Solicoccozyma gelidoterrea C23 B17 (OQ448362)
Solicoccozyma gelidoterrea C12 B02 (OQ448379)
Solicoccozyma gelidoterrea C23 B01 (OQ448347)
Solicoccozyma gelidoterrea CBS 9287 (MK397489)
Solicoccozyma gelidoterrea CBS 15580 (MK050340)
Solicoccozyma gelidoterrea CBS 9627 (KY105431)
Solicoccozyma gelidoterrea DBVPG:10727 (MK070335)
Solicoccozyma gelidoterrea C23 B05 (OQ448351)
Solicoccozyma gelidoterrea C07 B15 (OQ448338)
Solicoccozyma gelidoterrea K45 B02 (OQ448440)
Solicoccozyma gelidoterrea C23 B21 (OQ448364)
0.1
Solicoccozyma gelidoterrea C23 B15 (OQ448361)
